# Supplementary material for: Battery electric vehicles show the lowest carbon footprints among passenger cars across 1.5–3.0 °C energy decarbonisation pathways
Source: Commun Earth Environ. 2025 Jun 18;6(1):476. doi: 10.1038/s43247-025-02447-2 (PMC12176635; doi:10.1038/s43247-025-02447-2)
Supplement: Supplementary file 2 — Description of Additional Supplementary Files [file 43247_2025_2447_MOESM2_ESM.pdf]

Description of Additional Supplementary Files

File name: Supplementary Data 1
Description: Provides all underlying code, data, and results. The “1 Methods“ folder contains Python scripts and datasets required to generate inventories for future scenarios and passenger cars, and conduct prospective analysis. The “2 Results“ folder contains all figure datapoints.
