# Supplementary material for: Battery electric vehicles show the lowest carbon footprints among passenger cars across 1.5–3.0 °C energy decarbonisation pathways
Source: Commun Earth Environ. 2025 Jun 18;6(1):476. doi: 10.1038/s43247-025-02447-2 (PMC12176635; doi:10.1038/s43247-025-02447-2)
Supplement: Supplementary file 3 — Supplementary Data 1 [file 43247_2025_2447_MOESM3_ESM.zip › 1 Methods/0 Generate future scenarios.html]

0 Generate future scenarios


### Import libraries¶

In [ ]:

```
#!pip install matplotlib
#!pip install numpy==1.26.1 # Needed to downgrade numpy version
import time
import numpy as np
import pandas as pd
import bw2calc as bc
import bw2io as bi
import bw2data as bd
import os
from premise import *
from premise_gwp import add_premise_gwp
```

#### Set up: project, biosphere, ecoinvent (technosphere), and Premise-adjusted IPCC 2021 GWP method¶

The Premise-adjusted IPCC 2021 GWP method must be imported to account for negative biogenic carbon flows, such as those associated with BECCS technology, which is implemented in Premise.

In [ ]:

```
bd.projects.set_current("WP3_paper")
bi.bw2setup()

if 'ecoinvent 3.9.1' in bd.databases:
    print("Database has already been imported.")
else:
    #fp = r"C:\Users\js3700\PLCA-LIBs\ecoinvent 3.8\datasets"
    fp = r"C:\Users\js3700\EcoInvent\ecoinvent 3.9.1\datasets"
    ei = bi.SingleOutputEcospold2Importer(fp, 'ecoinvent 3.9.1')
    ei.apply_strategies()
    ei.statistics()
    ei.write_database()

add_premise_gwp()
```

In [ ]:

```
bd.projects.set_current("WP3_paper")
list(bd.databases)
```

#### Generate TIAM-ecoinvent pLCIs¶

The loop below generates scenarios sequentially, one at a time. This approach is necessary because processing multiple scenarios with multiple transformations simultaneously can lead to system instability or crashes.

In [ ]:

```
fp = "/Users/js3700/WP2-TIAM-premise/premise"  # Internal filepath that contains TIAM-UCL scenarios
pathways = ["SSP2-RCP60", "SSP2-RCP45", "SSP2-RCP26", "SSP2-RCP19"]
years = [2025, 2030, 2035, 2040, 2045, 2050]

total_start_time = time.time()

for pathway in pathways:
    for year in years:
        
        loop_start_time = time.time()
        
        scenarios = [{"model": "tiam-ucl", "pathway": pathway, "year": year, "filepath": fp}]
        ndb = NewDatabase(scenarios=scenarios, source_db='ecoinvent 3.9.1', source_version='3.9.1', use_absolute_efficiency=True)
        ndb.update(['heat', 'electricity', 'fuels', 'cement', 'steel', 'dac', 'biomass'])
        ndb.write_db_to_brightway()

        loop_end_time = time.time()
        loop_elapsed_time = (loop_end_time - loop_start_time) / 60
        print(f"Processed scenario: {scenarios} in {loop_elapsed_time:.2f} minutes")

total_end_time = time.time()
total_elapsed_time = (total_end_time - total_start_time) / 60
print(f"Total processing time: {total_elapsed_time:.2f} minutes")
```
