# Supplementary material for: Battery electric vehicles show the lowest carbon footprints among passenger cars across 1.5–3.0 °C energy decarbonisation pathways
Source: Commun Earth Environ. 2025 Jun 18;6(1):476. doi: 10.1038/s43247-025-02447-2 (PMC12176635; doi:10.1038/s43247-025-02447-2)
Supplement: Supplementary file 3 — Supplementary Data 1 [file 43247_2025_2447_MOESM3_ESM.zip › 1 Methods/1 Inventory creation .html]

1 Inventory creation 


# Import libraries¶

In [ ]:

```
import carculator
from carculator import *
import matplotlib.pyplot as plt
import time
import numpy as np
import pandas as pd
import bw2calc as bc
import bw2io as bi
import bw2data as bd
import seaborn as sns
import warnings
import random
import re
from datetime import datetime
from SALib.sample import saltelli
from SALib.analyze import sobol 
from SALib.sample import morris as ms
from SALib.analyze import morris as ma
from ipywidgets import widgets
from IPython.display import display
from tqdm.notebook import tqdm
from premise_gwp import add_premise_gwp

# Adding GWP information from the 'premise_gwp' package
add_premise_gwp()
```

# Set up bw2 project containing TIAM-UCL ecoinvents¶

In [ ]:

```
bd.projects
```

In [ ]:

```
bw.projects.set_current('WP3_paper')
bw.bw2setup()

#import ecoinvent 3.8
if 'ecoinvent 3.9.1' in bw.databases:
    print("Database has already been imported.")
else:
    #fp = r"C:\Users\js3700\PLCA-LIBs\ecoinvent 3.8\datasets"
    fp = r"C:\Users\js3700\EcoInvent\ecoinvent 3.9.1\datasets"
    ei = bw.SingleOutputEcospold2Importer(fp, 'ecoinvent 3.9.1')
    ei.apply_strategies()
    ei.statistics()
    ei.write_database()
```

# Generate carculator vehicle inventories¶

In [ ]:

```
#Trying to export all vehicles sizes
cip = CarInputParameters()
cip.static()
#cip.stochastic(2)

scope = {
    'powertrain':['FCEV', 'BEV', 'PHEV-d', 'HEV-d'], #all powertrains considered, only diesel for combustion...
    'year':[2020, 2030, 2040, 2050], #only year 2020 model for powertrain considered - 2020 < models consider efficiency improvements etc.
    'size':['Large','Large SUV','Lower medium','Medium','Medium SUV','Mini','Small'] #only biggest size of vehicle considered for now... 
}

batt_chem = {
    ("BEV", "Large", 2020): "NMC-811",
    ("BEV", "Large", 2030): "NMC-811",
    ("BEV", "Large", 2040): "NMC-811",
    ("BEV", "Large", 2050): "NMC-811",
    ("FCEV", "Large", 2020): "NMC-811",
    ("FCEV", "Large", 2030): "NMC-811",
    ("FCEV", "Large", 2040): "NMC-811",
    ("FCEV", "Large", 2050): "NMC-811",
    ("PHEV-d", "Large", 2020): "NMC-811",
    ("PHEV-d", "Large", 2030): "NMC-811",
    ("PHEV-d", "Large", 2040): "NMC-811",
    ("PHEV-d", "Large", 2050): "NMC-811",
    ("HEV-d", "Large", 2020): "NMC-811",
    ("HEV-d", "Large", 2030): "NMC-811",
    ("HEV-d", "Large", 2040): "NMC-811",
    ("HEV-d", "Large", 2050): "NMC-811",
 #   ("ICEV-d", "Large", 2020): "NMC-811",
 #   ("ICEV-d", "Large", 2030): "NMC-811",
 #   ("ICEV-d", "Large", 2040): "NMC-811",
 #   ("ICEV-d", "Large", 2050): "NMC-811",

    ("BEV", 'Large SUV', 2020): "NMC-811",
    ("BEV", 'Large SUV', 2030): "NMC-811",
    ("BEV", 'Large SUV', 2040): "NMC-811",
    ("BEV", 'Large SUV', 2050): "NMC-811",
    ("FCEV", 'Large SUV', 2020): "NMC-811",
    ("FCEV", 'Large SUV', 2030): "NMC-811",
    ("FCEV", 'Large SUV', 2040): "NMC-811",
    ("FCEV", 'Large SUV', 2050): "NMC-811",
    ("PHEV-d", 'Large SUV', 2020): "NMC-811",
    ("PHEV-d", 'Large SUV', 2030): "NMC-811",
    ("PHEV-d", 'Large SUV', 2040): "NMC-811",
    ("PHEV-d", 'Large SUV', 2050): "NMC-811",
    ("HEV-d", 'Large SUV', 2020): "NMC-811",
    ("HEV-d", 'Large SUV', 2030): "NMC-811",
    ("HEV-d", 'Large SUV', 2040): "NMC-811",
    ("HEV-d", 'Large SUV', 2050): "NMC-811",
   # ("ICEV-d", 'Large SUV', 2020): "NMC-811",
   # ("ICEV-d", 'Large SUV', 2030): "NMC-811",
   # ("ICEV-d", 'Large SUV', 2040): "NMC-811",
   # ("ICEV-d", 'Large SUV', 2050): "NMC-811",
    
    ("BEV", 'Lower medium', 2020): "NMC-811",
    ("BEV", 'Lower medium', 2030): "NMC-811",
    ("BEV", 'Lower medium', 2040): "NMC-811",
    ("BEV", 'Lower medium', 2050): "NMC-811",
    ("FCEV", 'Lower medium', 2020): "NMC-811",
    ("FCEV", 'Lower medium', 2030): "NMC-811",
    ("FCEV", 'Lower medium', 2040): "NMC-811",
    ("FCEV", 'Lower medium', 2050): "NMC-811",
    ("PHEV-d", 'Lower medium', 2020): "NMC-811",
    ("PHEV-d", 'Lower medium', 2030): "NMC-811",
    ("PHEV-d", 'Lower medium', 2040): "NMC-811",
    ("PHEV-d", 'Lower medium', 2050): "NMC-811",
    ("HEV-d", 'Lower medium', 2020): "NMC-811",
    ("HEV-d", 'Lower medium', 2030): "NMC-811",
    ("HEV-d", 'Lower medium', 2040): "NMC-811",
    ("HEV-d", 'Lower medium', 2050): "NMC-811",
 #   ("ICEV-d", 'Lower medium', 2020): "NMC-811",
 #   ("ICEV-d", 'Lower medium', 2030): "NMC-811",
 #   ("ICEV-d", 'Lower medium', 2040): "NMC-811",
 #   ("ICEV-d", 'Lower medium', 2050): "NMC-811",
    
    ("BEV", 'Medium', 2020): "NMC-811",
    ("BEV", 'Medium', 2030): "NMC-811",
    ("BEV", 'Medium', 2040): "NMC-811",
    ("BEV", 'Medium', 2050): "NMC-811",
    ("FCEV", 'Medium', 2020): "NMC-811",
    ("FCEV", 'Medium', 2030): "NMC-811",
    ("FCEV", 'Medium', 2040): "NMC-811",
    ("FCEV", 'Medium', 2050): "NMC-811",
    ("PHEV-d", 'Medium', 2020): "NMC-811",
    ("PHEV-d", 'Medium', 2030): "NMC-811",
    ("PHEV-d", 'Medium', 2040): "NMC-811",
    ("PHEV-d", 'Medium', 2050): "NMC-811",
    ("HEV-d", 'Medium', 2020): "NMC-811",
    ("HEV-d", 'Medium', 2030): "NMC-811",
    ("HEV-d", 'Medium', 2040): "NMC-811",
    ("HEV-d", 'Medium', 2050): "NMC-811",
#    ("ICEV-d", 'Medium', 2020): "NMC-811",
#    ("ICEV-d", 'Medium', 2030): "NMC-811",
#    ("ICEV-d", 'Medium', 2040): "NMC-811",
#    ("ICEV-d", 'Medium', 2050): "NMC-811",
    
    ("BEV", 'Medium SUV', 2020): "NMC-811",
    ("BEV", 'Medium SUV', 2030): "NMC-811",
    ("BEV", 'Medium SUV', 2040): "NMC-811",
    ("BEV", 'Medium SUV', 2050): "NMC-811",
    ("FCEV", 'Medium SUV', 2020): "NMC-811",
    ("FCEV", 'Medium SUV', 2030): "NMC-811",
    ("FCEV", 'Medium SUV', 2040): "NMC-811",
    ("FCEV", 'Medium SUV', 2050): "NMC-811",
    ("PHEV-d", 'Medium SUV', 2020): "NMC-811",
    ("PHEV-d", 'Medium SUV', 2030): "NMC-811",
    ("PHEV-d", 'Medium SUV', 2040): "NMC-811",
    ("PHEV-d", 'Medium SUV', 2050): "NMC-811",
    ("HEV-d", 'Medium SUV', 2020): "NMC-811",
    ("HEV-d", 'Medium SUV', 2030): "NMC-811",
    ("HEV-d", 'Medium SUV', 2040): "NMC-811",
    ("HEV-d", 'Medium SUV', 2050): "NMC-811",
#    ("ICEV-d", 'Medium SUV', 2020): "NMC-811",
#    ("ICEV-d", 'Medium SUV', 2030): "NMC-811",
#    ("ICEV-d", 'Medium SUV', 2040): "NMC-811",
#    ("ICEV-d", 'Medium SUV', 2050): "NMC-811",
    
    ("BEV", 'Mini', 2020): "NMC-811",
    ("BEV", 'Mini', 2030): "NMC-811",
    ("BEV", 'Mini', 2040): "NMC-811",
    ("BEV", 'Mini', 2050): "NMC-811",
    ("FCEV", 'Mini', 2020): "NMC-811",
    ("FCEV", 'Mini', 2030): "NMC-811",
    ("FCEV", 'Mini', 2040): "NMC-811",
    ("FCEV", 'Mini', 2050): "NMC-811",
    ("PHEV-d", 'Mini', 2020): "NMC-811",
    ("PHEV-d", 'Mini', 2030): "NMC-811",
    ("PHEV-d", 'Mini', 2040): "NMC-811",
    ("PHEV-d", 'Mini', 2050): "NMC-811",
    ("HEV-d", 'Mini', 2020): "NMC-811",
    ("HEV-d", 'Mini', 2030): "NMC-811",
    ("HEV-d", 'Mini', 2040): "NMC-811",
    ("HEV-d", 'Mini', 2050): "NMC-811",
#    ("ICEV-d", 'Mini', 2020): "NMC-811",
#    ("ICEV-d", 'Mini', 2030): "NMC-811",
#    ("ICEV-d", 'Mini', 2040): "NMC-811",
#    ("ICEV-d", 'Mini', 2050): "NMC-811",
    
    ("BEV", 'Small', 2020): "NMC-811",
    ("BEV", 'Small', 2030): "NMC-811",
    ("BEV", 'Small', 2040): "NMC-811",
    ("BEV", 'Small', 2050): "NMC-811",
    ("FCEV", 'Small', 2020): "NMC-811",
    ("FCEV", 'Small', 2030): "NMC-811",
    ("FCEV", 'Small', 2040): "NMC-811",
    ("FCEV", 'Small', 2050): "NMC-811",
    ("PHEV-d", 'Small', 2020): "NMC-811",
    ("PHEV-d", 'Small', 2030): "NMC-811",
    ("PHEV-d", 'Small', 2040): "NMC-811",
    ("PHEV-d", 'Small', 2050): "NMC-811",
    ("HEV-d", 'Small', 2020): "NMC-811",
    ("HEV-d", 'Small', 2030): "NMC-811",
    ("HEV-d", 'Small', 2040): "NMC-811",
    ("HEV-d", 'Small', 2050): "NMC-811",
#    ("ICEV-d", 'Small', 2020): "NMC-811",
#    ("ICEV-d", 'Small', 2030): "NMC-811",
#    ("ICEV-d", 'Small', 2040): "NMC-811",
#    ("ICEV-d", 'Small', 2050): "NMC-811",
    
}

dcts, array = fill_xarray_from_input_parameters(cip, scope=scope)
array.loc[dict(parameter="lifetime kilometers")] = 200000 #lifetime kilometres adjusted to 150,000 km instead of > 200,000 km..
cm = CarModel(array, cycle='WLTC', country='RER',energy_storage={"electric": batt_chem})
cm.set_all()

ic = InventoryCar(cm)

ic.export_lci(
    software="brightway2",
    directory=r"/Users/js3700/WP3-carculator-pLCA/carculator",
    ecoinvent_version='3.8',
    format="file"
)
```

# Functions to process carculator inventories¶

In [ ]:

```
def remove_noise_rows(filename):
    # Read the Excel file into a DataFrame
    df = pd.read_excel(filename)
    
    # Convert the first column to string
    df.iloc[:, 0] = df.iloc[:, 0].astype(str)
    
    # Remove rows where the first column starts with "noise, octave"
    df = df[~df.iloc[:, 0].str.startswith("noise, octave ")]
    
    # Replace 'nan' with blank spaces
    df.iloc[:, 0] = df.iloc[:, 0].replace('nan', '')
    
    return df

#In combine excel files, let's combine new market for hydrogen activity too Hydrogen, gaseous, 700 bar, from market, at fuelling station

def combine_excel_files(file1, file2, output_file):
    # Clean and read the first Excel file into a DataFrame
    df1 = remove_noise_rows(file1)
    
    # Read the second Excel file into another DataFrame
    df2 = pd.read_excel(file2)
    
    # Concatenate the two DataFrames
    combined_df = pd.concat([df1, df2], ignore_index=True)
    
    # Write the combined DataFrame to a new Excel file
    combined_df.to_excel(output_file, index=False)

#Here, we need to regionalise our new (1) Hydrogen activities (2) Market for hydrogen gaseous and (3) Replace H vehicle supply with new 

def regionalize_electricity_inputs_and_drop_unused_lci(file_path, region):
    
    # List of activities to remove, extend list further when doing cleaning later on...
    activities_to_remove = [
        'electricity supply', 
        'fuel supply for diesel',
        'fuel supply for hydrogen', 
        'transport, Kick Scooter, electric, <1kW, NMC battery, 2020',
        'transport, Bicycle',
        'transmission, for lorry',
        'tires and wheels, for lorry',
        'suspension, for lorry',
        'retarder, for lorry',
        'power electronics, for lorry',
        'other components, for hybrid electric lorry',
        'other components, for electric lorry',
        'methanol synthesis, hydrogen from electrolysis, CO2 from DAC',
        'methanol production facility, construction',
        'methanol distillation, hydrogen from electrolysis, CO2 from DAC',
        'maintenance, electric bicycle, without battery',
        'lead acid battery, for lorry',
        'internal combustion engine, for lorry',
        'gearbox, for lorry',
        'Ethanol',
        #'ATR',
        'Biodiesel',
        'Biomethane',
        'diesel',
        'Diesel',
        'Farming',
        'Extraction of vegetable',
        #'Fiber',
        'Methane',
        'Refining of vegetable',
        #'SMR',
        'Supply',
        'Syngas',
        'assembly operation, for lorry',
        'biogas',
        #'biomethane',
        'cabin, for lorry',
        'electric bicycle',
        'electric cargo bicycle',
        'electricity production, at',
        'frame, blanks and saddle, for lorry',
        'gasoline',
        'Crude Palm Oil',
        'Gas-to-liquid',
        'EV charger',
        'Storage battery',
        'Refining of crude vegetable oil from rapeseed',
    ]
    
    duplicates = [
        'Hydrogen, gaseous, 25 bar, from heatpipe reformer gasification of woody biomass, at gasification plant',
        'Hydrogen, gaseous, 25 bar, from heatpipe reformer gasification of woody biomass with CCS, at gasification plant',
        'Hydrogen, gaseous, 25 bar, from gasification of woody biomass in entrained flow gasifier, with CCS, at gasification plant',
        'Hydrogen, gaseous, 25 bar, from gasification of woody biomass in entrained flow gasifier, at gasification plant',
        'water, pure, via reverse osmosis',
        'water, pure, via reverse osmosis',
        'lithium carbonate precipitation 1',
        'lithium carbonate precipitation 2',
    ]
    
    # Read the excel file
    df = pd.read_excel(file_path, engine='openpyxl')
    rows_to_drop = []
    activities_to_regionalize_exchanges = []

    # Initialize a flag to determine if we're within an activity that starts with "Car, "
    in_car_activity = False
    remove_activity = False

    # Iterate through the rows of the DataFrame
    for idx, row in df.iterrows():

        #1. First, we change locations of the activities

        if row[0] == 'Activity':
            activities_to_regionalize_exchanges.append(df.at[idx, 1])

        if row[0] == 'location':
            df.at[idx, 1] = region
        
        #Check for an "Activity" starting with "Car, "
        if row[0] == 'Activity' and str(row[1]).startswith('Car, ') or str(row[1]).startswith('transport, car'):
            in_car_activity = True
        elif row[0] == 'Activity':
            in_car_activity = False

        #If we are in a "Car, " activity, modify the location to "CN"
        if in_car_activity and row[0] == 'location':
            df.at[idx, 1] = region

        # If this row starts a new activity, check if it's the one we want to remove
        if row[0] == 'Activity':
            if any(str(row[1]).startswith(activity) for activity in activities_to_remove):
                remove_activity = True
                rows_to_drop.append(idx)
            else:
                # If we were previously in the 'remove_activity' mode, 
                # this new activity means we are out of it now
                remove_activity = False
        
        # If we are in 'remove_activity' mode, continue appending rows to be dropped
        elif remove_activity:
            #if not pd.isna(row[0]):
            rows_to_drop.append(idx)

        #2. Next, we remove original carculator electricity inventories for use with ecoinvent ones
        if str(row[0]).startswith('electricity supply for battery production') or str(row[0]).startswith('electricity supply for electric vehicles'):
            df.at[idx, 0] = 'market group for electricity, low voltage'
            df.at[idx, 3] = region

        if str(row[0]).startswith('electricity supply for fuel preparation'):
            df.at[idx, 0] = 'market group for electricity, low voltage'
            df.at[idx, 3] = region

        if str(row[0]).startswith('fuel supply for diesel vehicles'):
            df.at[idx, 0] = 'market for diesel, low-sulfur' # this was original market group acitivty
            df.at[idx, 3] = region # this was original global..

        if str(row[0]).startswith('Car, ') or str(row[0]).startswith('transport, car'):
            df.at[idx, 3] = region

        if str(row[0]).startswith('market group for electricity, medium voltage') or str(row[0]).startswith('market for electricity, medium voltage'):
            df.at[idx, 0] = 'market group for electricity, medium voltage'
            df.at[idx, 3] = region

        if str(row[0]).startswith('market group for electricity, low voltage') or str(row[0]).startswith('market for electricity, low voltage'):
            df.at[idx, 0] = 'market group for electricity, low voltage'
            df.at[idx, 3] = region

        if str(row[0]).startswith('market group for electricity, high voltage') or str(row[0]).startswith('market for electricity, high voltage'):
            df.at[idx, 0] = 'market group for electricity, high voltage'
            df.at[idx, 3] = region

        #Attempt #1 for 'market for hydrogen, gaseous'
        if str(row[0]).startswith('market for hydrogen, gaseous'):
            df.at[idx, 0] = 'market for hydrogen, gaseous'
            df.at[idx, 3] = region

        #Attempt #2 for 'replacing the fuel supply for hydrogen vehicles
        if str(row[0]).startswith('fuel supply for hydrogen vehicles,'):
            df.at[idx, 0] = 'Hydrogen, gaseous, 700 bar, from market, at fuelling station'
            df.at[idx, 3] = region
            df.at[idx, 7] = 'Hydrogen, gaseous, 700 bar'

        #Attempt #3 for 'replacing the fuel supply for hydrogen vehicles
        if str(row[0]).startswith('Activity') and str(row[1]).startswith('Hydrogen, gaseous, 700 bar, from market, at fuelling station'):
            df.at[idx+1, 1] = region

        #changes for ecoinvent 3.9 compatibility

        if str(row[0]).startswith('NMVOC, non-methane volatile organic compounds, unspecified origin'):
            df.at[idx, 0] = 'NMVOC, non-methane volatile organic compounds'

        if str(row[0]).startswith('market for anode, graphite, for lithium-ion battery'):
            df.at[idx, 0] = 'market for anode, graphite, for Li-ion battery'
            df.at[idx, 3] = 'RoW'
            df.at[idx, 7] = 'anode, graphite, for Li-ion battery'

        if str(row[0]).startswith('market for concrete, normal') or str(row[0]).startswith('market for concrete, high exacting requirements') or str(row[0]).startswith('market for concrete, for de-icing salt contact'):
            df.at[idx, 0] = 'market for concrete, normal strength'
            df.at[idx, 7] = 'concrete, normal strength'

        if str(row[0]).startswith('treatment of wastewater, average, capacity 1E9l/year') or str(row[0]).startswith('treatment of wastewater, from residence, capacity 1.1E10l/year'):
            df.at[idx, 0] = 'treatment of wastewater, average, wastewater treatment'
            df.at[idx, 7] = 'wastewater, average'

        if str(row[0]).startswith('treatment of wastewater, unpolluted, capacity 5E9l/year'):
            df.at[idx, 0] = 'treatment of wastewater, unpolluted, wastewater treatment'

        #here need to use strip instead i think...
        
        if str(row[0]).startswith('Hydrogen chloride'):
            df.at[idx, 0] = 'Hydrochloric acid'

        if str(row[0]).startswith('Arsenic') or str(row[0]).startswith('Arsenic, ion'):
            df.at[idx, 0] = 'Arsenic ion'

        if str(row[0]).startswith('Cadmium'):
            df.at[idx, 0] = 'Cadmium II'

        if str(row[0]).strip() == 'Chromium':
            df.at[idx, 0] = 'Chromium III'

        if str(row[0]).strip() == 'Copper':
            df.at[idx, 0] = 'Copper ion'

        if str(row[0]).strip() == 'Copper, in ground':
            df.at[idx, 0] = 'Copper'

        if str(row[0]).strip() == 'Mercury':
            df.at[idx, 0] = 'Mercury II'

        if str(row[0]).strip() == 'Nickel':
            df.at[idx, 0] = 'Nickel II'

        if str(row[0]).strip() == 'Selenium':
            df.at[idx, 0] = 'Selenium IV'

        if str(row[0]).strip() == 'Zinc':
            df.at[idx, 0] = 'Zinc II'

        if str(row[0]).strip() == 'market for diesel, low-sulfur': # this was original market group for diesl,, just in case
            df.at[idx, 7] = 'diesel, low-sulfur'

        if str(row[0]).strip() == 'Ethene':
            df.at[idx, 0] = 'Ethylene'

        if str(row[0]).strip() == 'market group for electricity, medium voltage':
            df.at[idx, 7] = 'electricity, medium voltage'

        if str(row[0]).strip() == 'Cobalt, in ground':
            df.at[idx, 0] = 'Cobalt'

        if str(row[0]).strip() == 'Particulates, < 2.5 um':
            df.at[idx, 0] = 'Particulate Matter, < 2.5 um'

        if str(row[0]).strip() == 'Particulates, > 2.5 um, and < 10um':
            df.at[idx, 0] = 'Particulate Matter, > 2.5 um and < 10um'

        if str(row[0]).strip() == 'Oil, crude, in ground':
            df.at[idx, 0] = 'Oil, crude'

        if str(row[0]).strip() == 'AOX, Adsorbable Organic Halogen as Cl':
            df.at[idx, 0] = 'AOX, Adsorbable Organic Halogen'

        if str(row[0]).strip() == 'Aluminium':
            df.at[idx, 0] = 'Aluminium III'

        if str(row[0]).strip() == 'Barium':
            df.at[idx, 0] = 'Barium II'

        if str(row[0]).strip() == 'Calcium, ion':
            df.at[idx, 0] = 'Calcium II'

        if str(row[0]).strip() == 'Chromium, ion':
            df.at[idx, 0] = 'Chromium III'

        if str(row[0]).strip() == 'Manganese':
            df.at[idx, 0] = 'Manganese II'

        if str(row[0]).strip() == 'Particulates, > 10 um':
            df.at[idx, 0] = 'Particulate Matter, > 10 um'

        if str(row[0]).strip() == 'Potassium, ion':
            df.at[idx, 0] = 'Potassium I'

        if str(row[0]).strip() == 'Sodium, ion':
            df.at[idx, 0] = 'Sodium I'

        if str(row[0]).strip() == 'Zinc, ion':
            df.at[idx, 0] = 'Zinc II'

        if str(row[0]).strip() == 'electricity, high voltage, production mix':
            df.at[idx, 3] = 'CN-CSG'

        if str(row[0]).strip() == 'Lithium, in ground':
            df.at[idx, 0] = 'Lithium'

        if str(row[0]).strip() == 'market for soda ash, light, crystalline, heptahydrate':
            df.at[idx, 0] = 'market for soda ash, light'
            df.at[idx, 7] = 'soda ash, light'

        if str(row[0]).strip() == 'Iron, ion':
            df.at[idx, 0] = 'Iron ion'

        if str(row[0]).strip() == 'Lithium, ion':
            df.at[idx, 0] = 'Lithium I'

    for idx, row in df.iterrows():
        if row[0] in activities_to_regionalize_exchanges:
            df.at[idx, 3] = region

    removing_duplicates = False

    for idx, row in df.iterrows():
        if row[0] == 'Activity':
            if str(row[1]) in duplicates:
                removing_duplicates = True
                rows_to_drop.append(idx)
                duplicates.remove(str(row[1]))
            else:
                removing_duplicates = False
        elif removing_duplicates:
            rows_to_drop.append(idx)

    # Save the modified dataframe back to the same Excel file
    df.drop(rows_to_drop, inplace=True)
    df.to_excel(f"/Users/js3700/WP3-carculator-pLCA/LCI_COMBINED/regionalized_recycling_carculator_file.xlsx", index=False, engine='openpyxl')

#ADDS END OF LIFE ACTIVITIES TO CAR INVENTORIES AND SEPERATES OUT LIFE CYCLES
def create_end_of_life_activities(file_path):
    
    # Read the excel file
    df = pd.read_excel(file_path, engine='openpyxl')
    
    activity_names = []
    #sizes=['Large','Large SUV','Lower medium','Medium','Medium SUV','Mini','Small','Van']
    sizes=['Large','Large SUV','Lower medium','Medium','Medium SUV','Mini','Small']

    for size in sizes:
        cars = [
            f"transport, car, battery electric, NMC-811 battery, {size}, 2020",
            f"transport, car, battery electric, NMC-811 battery, {size}, 2030",
            f"transport, car, battery electric, NMC-811 battery, {size}, 2040",
            f"transport, car, battery electric, NMC-811 battery, {size}, 2050",
            
            f"transport, car, fuel cell electric, {size}, 2020",
            f"transport, car, fuel cell electric, {size}, 2030",
            f"transport, car, fuel cell electric, {size}, 2040",
            f"transport, car, fuel cell electric, {size}, 2050",
            
            f'transport, car, plugin diesel hybrid, {size}, 2020, Euro-6.2',
            f'transport, car, plugin diesel hybrid, {size}, 2030, Euro-7',
            f'transport, car, plugin diesel hybrid, {size}, 2040, Euro-7',
            f'transport, car, plugin diesel hybrid, {size}, 2050, Euro-7',
            
            f'transport, car, diesel hybrid, {size}, 2020, Euro-6.2',
            f'transport, car, diesel hybrid, {size}, 2030, Euro-7',
            f'transport, car, diesel hybrid, {size}, 2040, Euro-7',
            f'transport, car, diesel hybrid, {size}, 2050, Euro-7',
        
      #      f'transport, car, diesel, {size}, 2020, Euro-6.2',
      #      f'transport, car, diesel, {size}, 2030, Euro-7',
      #      f'transport, car, diesel, {size}, 2040, Euro-7',
      #      f'transport, car, diesel, {size}, 2050, Euro-7',
        ]
        for car in cars:
            activity_names.append(str(car))
        
    recycling_names = [
        "Recycling of NMC-811 battery cell, inorganic hydrometallurgical",
        "Recycling of NMC-811 battery cell, pyrometallurgical recycling",
        "Recycling of NMC-811 battery cell, direct recycling"
    ]

    #The below loops the different activity_names (vehicles) inventories that will be amended with each of the recycling_names...

    indices_to_drop = []
    
    for activity_name in activity_names:
        print(activity_name)
        for recycling_name in recycling_names:
            
            #Here, we are creating the end-of-life activities associated to each recycling type and vehicle type.
            activity_parts = activity_name.split(',')
            car_parts = activity_name.split(", ", 1)
            car_name = car_parts[1][:1].capitalize() + car_parts[1][1:]
            activity_parts[0] = "end-of-life, " + recycling_name.split(', ')[1]
            new_activity_name = ','.join(activity_parts).strip()
            activity_rows = df[df.iloc[:, 1] == activity_name]
            activity_row_num = df[df[1] == activity_name].index[0]
            activity_location = df.loc[activity_row_num + 1, 1]
            
            # For the activity above this adds the necessary data
            new_activity_data = [
                [None, None],
                ["Activity", new_activity_name],
                ["location", activity_location],
                ["production amount", 1],
                ["reference product", "Car, disposed"],
                ["type", "process"],
                ["unit", "unit"],
                ["worksheet name", None],
                ["source", None],
                ["description", None],
                ["special remark", None],
                ["comment", None],
                ["Exchanges", None],
                ["name", "amount", "database", "location", "unit", "categories", "type", "reference product", "tag"]
            ]

            #Adds the activity data without the exchanges below...
            df = pd.concat([df, pd.DataFrame(new_activity_data)], ignore_index=True)

            #Finding the battery amount that will be used as exchange data...
            find_battery_amount = (df.iloc[activity_row_num:] == 'Battery cell, NMC-811').idxmax()[0]
            battery_exchange_amount = df.loc[find_battery_amount, 1]

            #Adds the production exchange based on the battery amount...
            exchanges = [
                [new_activity_name, 1, "carculator_lci_car", activity_location, "unit", None, "production", "Car, disposed", "other"],
                [recycling_name, battery_exchange_amount, "carculator_lci_car", activity_location, "kg", None, "technosphere", "Battery cell", "other"]
            ]

            #But we also want to migrate some exchanges from the original activities
            exchanges_to_migrate = [
                "market for used powertrain from electric passenger car, manual dismantling", 
                "treatment of used glider, passenger car, shredding", 
                "treatment of used internal combustion engine, passenger car, shredding",
                "market for used Li-ion battery"
            ]

            # to keep track of which exchanges have been found for migration
            found_exchanges_migrate = set()  
            #indices_to_drop = []
            
            for index, row in df.iloc[activity_row_num:].iterrows():
                # Check for exchanges to migrate
                if row[0] in exchanges_to_migrate and row[0] not in found_exchanges_migrate:
                    # Update the amount for the "market for used Li-ion battery" row
                    #if row[0] == "market for used Li-ion battery":
                        #row[1] += battery_exchange_amount
                    exchanges.append(row.tolist())
                    found_exchanges_migrate.add(row[0])
                    indices_to_drop.append(index)
                
                # Exit condition
                if len(found_exchanges_migrate) == len(exchanges_to_migrate):
                    break

            exchanges = pd.DataFrame(exchanges)
            
            for idx, row in exchanges.iterrows():
                if row[0] == "market for used Li-ion battery":
                    exchanges.at[idx, 1] += battery_exchange_amount
            
            # Drop the rows from the original DataFrame
            #df.drop(indices_to_drop, inplace=True)

            df = pd.concat([df, exchanges], ignore_index=True)
            #df = pd.concat([df, pd.DataFrame(exchanges)], ignore_index=True)

            #Now I'm attempting to create new vehicle activity based on the recycling process it takes...
            new_car_data = [
                [None, None],
                ["Activity", activity_name + ', ' + recycling_name.split(', ')[1]],
                ["location", activity_location],
                ["production amount", 1],
                ["reference product", "transport, car"],
                ["type", "process"],
                ["unit", "kilometer"],
                ["worksheet name", None],
                ["source", None],
                ["description", None],
                ["special remark", None],
                ["comment", None],
                ["Exchanges", None],
                ["name", "amount", "database", "location", "unit", "categories", "type", "reference product", "tag"]
            ]

            #Adds the activity data without the exchanges below...
            df = pd.concat([df, pd.DataFrame(new_car_data)], ignore_index=True)

            new_car_data_exchanges = [
                [activity_name + ', ' + recycling_name.split(', ')[1], 1, "carculator_lci_car", activity_location, "kilometer", None, "production", "transport, car", "other"],
                #[recycling_name, battery_exchange_amount, "carculator_lci_car", "GLO", "kg", None, "technosphere", "Battery cell", "other"]
            ]

            avoid_these = ['location','production amount','reference product','type','unit','worksheet name','source','Exchanges','name']
            
            for index, row in df.iloc[activity_row_num+1:].iterrows():
                if row[0] == "Activity":
                    break
                
                if row[0] == car_name:
                    x = row[1]
                    new_row_car_data = [new_activity_name, x, "carculator_lci_car", activity_location, "unit", None, "technosphere", "Car, disposed", None]
                    new_car_data_exchanges.append(new_row_car_data)
                
                if row[0] not in exchanges_to_migrate and row[0] != activity_name and row[0] not in avoid_these:
                    new_car_data_exchanges.append(row.tolist())
            
            df = pd.concat([df, pd.DataFrame(new_car_data_exchanges)], ignore_index=True)

    df.drop(indices_to_drop, inplace=True)
    
    #print(exchanges)

    df = df.loc[df.iloc[:, 0].astype(str) != 'comment']
    df = df.loc[df.iloc[:, 0].astype(str) != 'special remark']
    df = df.loc[df.iloc[:, 0].astype(str) != 'description']

    #Now we need to remove the original activities...
    rows_to_drop = []
    remove_activity = False

    for idx, row in df.iterrows():

        # If this row starts a new activity, check if it's the one we want to remove
        if row[0] == 'Activity':
            if any(str(row[1]) == activity for activity in activity_names):
            #if any(str(row[1]).startswith(activity) for activity in activity_names):
                remove_activity = True
                rows_to_drop.append(idx)
            else:
                # If we were previously in the 'remove_activity' mode, 
                # this new activity means we are out of it now
                remove_activity = False
        
        # If we are in 'remove_activity' mode, continue appending rows to be dropped
        elif remove_activity:
            #if not pd.isna(row[0]):
            rows_to_drop.append(idx)

    #We also need to 

    df.drop(rows_to_drop, inplace=True)
    df.to_excel(f"/Users/js3700/WP3-carculator-pLCA/LCI_COMBINED/{region}_EOL_recycling_carculator_file.xlsx", index=False, engine='openpyxl')
    #print(activity_rows)

def process_excel_files(folder_path, years, scenarios, base_file_name='LCI_foreground.xlsx'):
    # Task 1: Combining multiple Excel sheets
    all_files = [f for f in os.listdir(folder_path) if f.endswith('_EOL_recycling_carculator_file.xlsx')]
    all_dataframes = [pd.read_excel(os.path.join(folder_path, file), engine='openpyxl', skiprows=2) for file in all_files]
    combined_df = pd.concat(all_dataframes, ignore_index=True)

    # Task 2: Drop first row, add new rows at the beginning
    combined_df.drop(combined_df.index[0], inplace=True)
    new_data = {
        combined_df.columns[0]: ['Database', 'format', ''],
        combined_df.columns[1]: ['LCI_CAN_RCP19_2020', 'Excel spreadsheet', ''],
        **{col: ['', '', ''] for col in combined_df.columns[2:]}
    }
    combined_df = pd.concat([pd.DataFrame(new_data), combined_df], ignore_index=True)

    # Task 3: Drop header
    combined_df.columns = range(combined_df.shape[1])

    # Write to base file
    combined_df.to_excel(os.path.join(folder_path, base_file_name), header=False, index=False, engine='openpyxl')

    # Task 4: Modify and write files for each scenario and year
    df = combined_df.copy()  # Load the base Excel data
    for year in years:
        for scenario in scenarios:
            new_file_name = f'LCI_foreground_{scenario}_{year}.xlsx'
            df.iloc[0, 1] = f'LCI_foreground_{scenario}_{year}'
            df.to_excel(os.path.join(folder_path, new_file_name), header=False, index=False, engine='openpyxl')
```

In [ ]:

```
regions = ['AFR','AUS','CAN','CHI','CSA','EEU','FSU','IND','JPN','MEA','MEX','ODA','SKO','UK','WEU','USA']
#regions = ['AFR']
file1_path = r"/Users/js3700/WP3-carculator-pLCA/carculator/carculator_lci_bw2.xlsx"
file2_path = r"/Users/js3700/WP3-carculator-pLCA/recycling_lci_complete_cutoff.xlsx"

for region in regions:

    folder_path = f"/Users/js3700/WP3-carculator-pLCA/LCI_COMBINED"
    
    # Check if the folder already exists or not
    if not os.path.exists(folder_path):
        # Create the new folder
        os.makedirs(folder_path)
        print(f"Folder '{folder_path}' created successfully.")
    else:
        print(f"Folder '{folder_path}' already exists.")
    
    output_file_path = f"/Users/js3700/WP3-carculator-pLCA/LCI_COMBINED/recycling_carculator_file.xlsx"
    combine_excel_files(file1_path, file2_path, output_file_path)
    
    file_path = output_file_path
    
    regionalize_electricity_inputs_and_drop_unused_lci(file_path, region)
    
    file_path = f"/Users/js3700/WP3-carculator-pLCA/LCI_COMBINED/regionalized_recycling_carculator_file.xlsx"
    create_end_of_life_activities(file_path)

# Call the function
folder_path = "/Users/js3700/WP3-carculator-pLCA/LCI_COMBINED/"
years = [2025,2030,2035,2040,2045,2050]
scenarios = ['RCP19','RCP26','RCP45','RCP60']
process_excel_files(folder_path, years, scenarios)
```

# Import and match generated carculator inventories to each version of TIAM-UCL¶

In [ ]:

```
def import_and_match(rcp_scenario, year, start_time):
    database_name = f"LCI_foreground_RCP{rcp_scenario}_{year}"
    if database_name in bd.databases:
        print(f"Database {database_name} has already been imported.")
        return

    print(f'FOUND:{database_name}')

    fp = rf"\Users\js3700\WP3-carculator-pLCA\LCI_COMBINED\{database_name}.xlsx"
    print(f'IMPORTING:{database_name}')
    i = bi.ExcelImporter(fp)
    i.apply_strategies()
    i.match_database(fields=["name", "unit", "location"])
    print(f'IMPORTED')

    # Find the exact matching database name in the current databases
    matching_db_name = None
    for db_name in bd.databases:
        if f"ecoinvent_cutoff_3.9_tiam-ucl_SSP2-RCP{rcp_scenario}_{year}" in db_name:
            matching_db_name = db_name
            break

    if matching_db_name:
        print(f'MATCHING TO:{matching_db_name}')
        i.match_database(matching_db_name, fields=["reference product", "name", "unit", "location"])
    else:
        print(f"No matching database found for ecoinvent_cutoff_3.9_tiam-ucl_SSP2-RCP{rcp_scenario}_{year}")

    i.statistics()
    i.write_database()
    print(f'COMPLETE')
    
    # Time update after each complete
    current_time = time.time()
    elapsed_time_minutes = (current_time - start_time) / 60
    print(f"Elapsed time: {elapsed_time_minutes:.2f} minutes")
    i.write_excel()

rcp_scenarios = [60, 45, 26, 19]
years = list(range(2025, 2055, 5))

start_time = time.time()

for rcp in rcp_scenarios:
    for year in years:
        import_and_match(rcp, year, start_time)
        time.sleep(1)

end_time = time.time()
total_elapsed_time_minutes = (end_time - start_time) / 60
print(f"Total time taken: {total_elapsed_time_minutes:.2f} minutes")
```
