# Supplementary material for: Battery electric vehicles show the lowest carbon footprints among passenger cars across 1.5–3.0 °C energy decarbonisation pathways
Source: Commun Earth Environ. 2025 Jun 18;6(1):476. doi: 10.1038/s43247-025-02447-2 (PMC12176635; doi:10.1038/s43247-025-02447-2)
Supplement: Supplementary file 3 — Supplementary Data 1 [file 43247_2025_2447_MOESM3_ESM.zip › 1 Methods/2 Analysis.html]

2 Analysis


## Import needed libraries¶

In [38]:

```
import time
import numpy as np
import pandas as pd
import bw2calc as bc
import bw2io as bi
import bw2data as bd
import matplotlib.pyplot as plt
import seaborn as sns
import warnings
from SALib.sample import saltelli
from SALib.analyze import sobol 
from SALib.sample import morris as ms
from SALib.analyze import morris as ma
import re
from datetime import datetime
from ipywidgets import widgets
from IPython.display import display
from tqdm.notebook import tqdm
from matplotlib.colors import Normalize
import glob
#from premise import *
#from premise_gwp import add_premise_gwp
bd.projects.set_current('WP3_paper')
```

# 0 Time-adjusted pLCA functions¶

In [ ]:

```
#PLCA Main Code - Attempting to speed things up
def get_databases_for_scenario(scenario, year, start_year, location):

    if 'No Scenario' in str(scenario):
        return [db for db in bd.databases if "LCI_foreground_RCP60" in db and str(year if year != start_year else start_year) in db]
    else:
        return [db for db in bd.databases if "LCI_foreground" in db and scenario in db and str(year if year != start_year else start_year) in db]

def compute_lca_score(FU, method):
    lca = bc.LCA(FU, method)
    lca.lci()
    lca.lcia()
    return lca.score

def compute_for_year_df(year, scenario, lifetime, method, region, vehicle, size, lifecycle, lifecycle_per_step, start_year, end_year, system_expansion, results_df, case, location):
    
    databases = get_databases_for_scenario(scenario, year, start_year, location)

    if 'No Scenario' in str(scenario):
            
        for database in databases:
            car = [x for x in bd.Database("LCI_foreground_RCP60_2025") if vehicle['name'] in x['name'] and vehicle['location'] in x['location']][0]
            production, eol = 0, 0  # default values
    
            for exc in car.technosphere():
                if "Car," in exc['name']:
                    production = compute_lca_score({exc.input: lifecycle * exc['amount']}, method)
      
                elif "end-of-life" in exc['name']:
                    eol = compute_lca_score({exc.input: lifecycle * exc['amount']}, method)
    
            if year == start_year:
                eol_score = eol * lifecycle_per_step
                df_production = pd.DataFrame({'Scenario': [scenario], 'Start':[start_year], 'Lifetime':[lifetime], 'Region': [region], 'Method': [method[1]], 'Vehicle': [vehicle['name']],'Size': [size], 'Total Mileage':[case], 'Year': [start_year], 'Type': ['Production'], 'Value': [production]})
                results_df = pd.concat([results_df, df_production])       
    
            total_use = ((compute_lca_score({car: lifecycle}, method) - production - eol) * (case/lifecycle) ) / lifecycle_per_step #the FU was case but realised not correct
            
            df_use = pd.DataFrame({'Scenario': [scenario], 'Start':[start_year], 'Lifetime':[lifetime],'Region': [region], 'Method': [method[1]], 'Vehicle': [vehicle['name']],'Size': [size], 'Total Mileage':[case], 'Year': [year], 'Type': ['Use'], 'Value': [total_use]})
            results_df = pd.concat([results_df, df_use])
            
            if year == end_year:
                eol_score = eol
                df_eol = pd.DataFrame({'Scenario': [scenario], 'Start':[start_year], 'Lifetime':[lifetime],'Region': [region],'Method': [method[1]], 'Vehicle': [vehicle['name']],'Size': [size], 'Total Mileage':[case], 'Year': [system_expansion], 'Type': ['End-of-life'], 'Value': [eol_score]})
                results_df = pd.concat([results_df, df_eol])
                
        return results_df

    else:
            
        for database in databases:
            car = [x for x in bd.Database(database) if vehicle['name'] in x['name'] and vehicle['location'] in x['location']][0]
            production, eol = 0, 0  # default values
    
            for exc in car.technosphere():
                if "Car," in exc['name']:
                    production = compute_lca_score({exc.input: lifecycle * exc['amount']}, method)
      
                elif "end-of-life" in exc['name']: # identify the end-of-life exchange

                    eol = compute_lca_score({exc.input: lifecycle * exc['amount']}, method) # calculate its score.
    
            if year == start_year:
                #eol_score = eol * lifecycle_per_step ###??????
                df_production = pd.DataFrame({'Scenario': [scenario], 'Start':[start_year],'Lifetime':[lifetime], 'Region': [region], 'Method': [method[1]], 'Vehicle': [vehicle['name']],'Size': [size], 'Total Mileage':[case], 'Year': [start_year], 'Type': ['Production'], 'Value': [production]})
                results_df = pd.concat([results_df, df_production])       
    
            total_use = ((compute_lca_score({car: lifecycle}, method) - production - eol) * (case/lifecycle) ) / lifecycle_per_step #the FU was case but realised not correct
            
            df_use = pd.DataFrame({'Scenario': [scenario], 'Start':[start_year],'Lifetime':[lifetime], 'Region': [region], 'Method': [method[1]], 'Vehicle': [vehicle['name']],'Size': [size], 'Total Mileage':[case], 'Year': [year], 'Type': ['Use'], 'Value': [total_use]})
            results_df = pd.concat([results_df, df_use])
            
            if year == end_year:
                eol_score = eol
                df_eol = pd.DataFrame({'Scenario': [scenario], 'Start':[start_year],'Lifetime':[lifetime], 'Region': [region],'Method': [method[1]], 'Vehicle': [vehicle['name']],'Size': [size], 'Total Mileage':[case], 'Year': [system_expansion], 'Type': ['End-of-life'], 'Value': [eol_score]})
                results_df = pd.concat([results_df, df_eol])
                
        return results_df

def PLCA(vehicle, size, lifetime, method, scenario, region, start_year, end_year, system_expansion, step, lifecycle, case, location):
    
    middle_years = [start_year + x for x in range(end_year-start_year) if x % 5 == 0][1:] + [end_year]
    lifecycle_per_step = ((end_year-start_year) / step)
    column_names=['Scenario', 'Start', 'Lifetime', 'Region', 'Method', 'Vehicle', 'Size', 'Total Mileage', 'Year', 'Type', 'Value']
    
    results_df = pd.DataFrame(columns=column_names)

    results_df = compute_for_year_df(start_year, scenario, lifetime, method, region, vehicle, size, lifecycle, lifecycle_per_step, start_year, end_year, system_expansion, results_df, case, location)
    for year in middle_years:
        results_df = compute_for_year_df(year, scenario, lifetime, method, region, vehicle, size, lifecycle, lifecycle_per_step, start_year, end_year, system_expansion, results_df, case, location)
            
    return results_df

def compute_cumulative(input_df, start_year):

    df = input_df.sort_values(by=["Scenario", 'Start', "Region", "Method", "Vehicle","Size", "Total Mileage", "Year"])
    new_data = {
        'Scenario': [],
        'Start':[],
        'Lifetime':[],
        'Region':[],
        'Method': [],
        'Vehicle': [],
        'Size':[],
        'Total Mileage':[],
        'Year': [],
        'Type': [],
        'Cumulative Value': []
    }
    
    cumulative_value = df.iloc[0]['Value']
    new_data['Scenario'].append(df.iloc[0]['Scenario'])
    new_data['Start'].append(df.iloc[0]['Start'])
    new_data['Lifetime'].append(df.iloc[0]['Lifetime'])
    new_data['Region'].append(df.iloc[0]['Region'])
    new_data['Method'].append(df.iloc[0]['Method'])
    new_data['Vehicle'].append(df.iloc[0]['Vehicle'])
    new_data['Size'].append(df.iloc[0]['Size'])
    new_data['Total Mileage'].append(df.iloc[0]['Total Mileage'])
    new_data['Year'].append(start_year)
    new_data['Type'].append('Production')
    new_data['Cumulative Value'].append(cumulative_value)
    
    previous_value = df.iloc[1]['Value']
    
    for i in range(2, len(df)):
        new_data['Scenario'].append(df.iloc[i]['Scenario'])
        new_data['Start'].append(df.iloc[i]['Start'])
        new_data['Lifetime'].append(df.iloc[i]['Lifetime'])
        new_data['Region'].append(df.iloc[i]['Region']) #why was this 0 and not i
        new_data['Method'].append(df.iloc[i]['Method'])
        new_data['Vehicle'].append(df.iloc[i]['Vehicle'])
        new_data['Size'].append(df.iloc[i]['Size'])
        new_data['Total Mileage'].append(df.iloc[i]['Total Mileage'])
        new_data['Year'].append(df.iloc[i]['Year'])
        new_data['Type'].append(df.iloc[i]['Type'])
        
        if df.iloc[i]['Type'] == 'Use':
            cumulative_value += (previous_value + df.iloc[i]['Value']) / 2
        else:
            cumulative_value += df.iloc[i]['Value']

        new_data['Cumulative Value'].append(cumulative_value)
        previous_value = df.iloc[i]['Value']

    new_df = pd.DataFrame(new_data)
    return new_df

def nearest_available_year(year):
    """
    Returns the nearest available year for vehicle data.
    Assumes available years are [2020, 2030, 2040, 2050].
    """
    if year <= 2025:
        return 2020
    elif 2025 < year <= 2035:
        return 2030
    elif 2035 < year <= 2045:
        return 2040
    else:
        return 2050
```

# 1 Comparative Monte Carlo carbon footprints¶

### Functions to run¶

In [ ]:

```
def monte_carlo_simulation(scenarios, regions, start_years, lifetimes, recycling_methods, sizes, powertrains, method, db_data, mileage_ranges, num_iterations=1, save_to_excel=True):
    """
    Perform a Monte Carlo simulation for vehicle LCA.
    
    Parameters:
        scenarios (list): List of scenarios.
        regions (list): List of regions.
        start_years (list): List of start years.
        lifetimes (list): List of lifetimes.
        recycling_methods (list): List of recycling methods.
        sizes (list): List of vehicle sizes.
        powertrains (list): List of powertrains.
        method (str): LCA method.
        db_data (list): Database of LCI data.
        mileage_ranges (list of tuples): List of mileage ranges (min, max) for random selection.
        num_iterations (int): Number of iterations for the simulation.
        save_to_excel (bool): Whether to save the results to an Excel file.
        
    Returns:
        pd.DataFrame: Results of the simulation.
    """
    start_time = time.time()
    warnings.simplefilter(action='ignore', category=FutureWarning)
    results_list = []

    def select_unique_size(sizes):
        """Select a unique size and return a regex pattern that matches only the exact size."""
        selected_size = random.choice(sizes)
        exact_size_pattern = rf'\b{re.escape(selected_size)}(?!\s\w)'
        return selected_size, exact_size_pattern

    for i in tqdm(range(num_iterations), desc="Running Simulations", leave=True, bar_format='{l_bar}{bar:30}{r_bar}{bar:-30b}'):
        iteration_start_time = time.time()

        selected_scenario = random.choice(scenarios)
        selected_region = random.choice(regions)
        selected_start_year = random.choice(start_years)
        selected_lifetime = random.choice(lifetimes)
        selected_recycling_method = random.choice(recycling_methods)
        selected_size, exact_size_pattern = select_unique_size(sizes)
        
        # Select a random mileage within the specified ranges
        selected_mileage_range = random.choice(mileage_ranges)
        selected_mileages = random.randint(selected_mileage_range[0], selected_mileage_range[1])

        # Determine the appropriate year for the vehicle data based on the selected start year
        if 2020 <= selected_start_year <= 2025:
            vehicle_year = '2020'
        elif 2025 <= selected_start_year <= 2030:
            vehicle_year = '2025'
        elif 2030 <= selected_start_year <= 2035:
            vehicle_year = '2030'
        elif 2035 <= selected_start_year <= 2040:
            vehicle_year = '2035'
        elif 2040 <= selected_start_year <= 2045:
            vehicle_year = '2040'
        elif 2045 <= selected_start_year <= 2050:
            vehicle_year = '2045'
        else:
            vehicle_year = '2050'

        vehicles = []
        
        for vehicle_type in powertrains:
            vehicle = next((x for x in db_data if 
                           f"transport, car, {vehicle_type}" in x['name'] and
                           re.search(exact_size_pattern, x['name']) and
                           selected_recycling_method in x['name'] and
                           vehicle_year in x['name'] and
                           selected_region in x['location']), None)
            if vehicle:
                vehicles.append(vehicle)

        for vehicle in vehicles:
            df_vehicle_scenario = compute_cumulative(PLCA(vehicle, selected_size, selected_lifetime, method, selected_scenario, selected_region, start_year=selected_start_year, end_year=(selected_start_year + selected_lifetime), system_expansion=(selected_start_year + selected_lifetime + 1), step=5, lifecycle=200000, case=selected_mileages, location=selected_region), selected_start_year)
            df_vehicle_scenario = df_vehicle_scenario[df_vehicle_scenario['Type'] == 'End-of-life']
            df_vehicle_scenario['Iteration'] = i + 1
            results_list.append(df_vehicle_scenario)

        iteration_end_time = time.time()

    results = pd.concat(results_list, ignore_index=True)
    df = results
    df.loc[df['Vehicle'].str.contains('transport, car, battery electric,', case=True, na=False), 'Vehicle'] = 'BEV'
    df.loc[df['Vehicle'].str.contains('transport, car, diesel hybrid,', case=True, na=False), 'Vehicle'] = 'HEV'
    df.loc[df['Vehicle'].str.contains('transport, car, fuel cell electric,', case=True, na=False), 'Vehicle'] = 'FCEV'
    df.loc[df['Vehicle'].str.contains('transport, car, plugin diesel hybrid,', case=True, na=False), 'Vehicle'] = 'PHEV'
    end_time = time.time()

    # Save the Excel file if required
    if save_to_excel:
        now = datetime.now()
        timestamp = now.strftime("%Y%m%d_%H%M%S")
        df.to_excel(f'MC_Final_{timestamp}.xlsx', index=False)

    #print(df)
    print(f"Execution time: {(end_time - start_time)/3600} hours")
    return df
```

#### Run for 2025 (current)¶

In [ ]:

```
# Parameters
scenarios = ['No Scenario','RCP60','RCP45','RCP26','RCP19']
regions = ['AFR', 'AUS', 'CAN', 'CHI', 'CSA', 'EEU', 'FSU', 'IND', 'JPN', 'MEA', 'MEX', 'ODA', 'SKO', 'UK', 'WEU', 'USA']
start_years = [2025]
lifetimes = [10, 15, 20]
recycling_methods = ['pyrometallurgical', 'inorganic hydrometallurgical', 'direct recycling']
sizes = ['Large', 'Large SUV', 'Lower medium', 'Medium', 'Medium SUV', 'Mini', 'Small']
powertrains = ['battery electric','diesel hybrid','fuel cell electric','plugin diesel hybrid']
method = [m for m in bd.methods if 'IPCC 2021' in str(m) and 'climate change' in str(m) and 'GWP 100a, incl. H and bio CO2' in str(m)][0]
db_data = list(bd.Database(f'LCI_foreground_RCP60_2025'))
mileage_ranges = [(100000, 300000)]

# Number of iterations
num_iterations = 5000

# Run the simulation
results_df = monte_carlo_simulation(scenarios, regions, start_years, lifetimes, recycling_methods, sizes, powertrains, method, db_data, mileage_ranges, num_iterations=num_iterations, save_to_excel=True)
```

#### Run for 2035 (future)¶

In [ ]:

```
# Parameters
scenarios = ['No Scenario','RCP60','RCP45','RCP26','RCP19']
regions = ['AFR', 'AUS', 'CAN', 'CHI', 'CSA', 'EEU', 'FSU', 'IND', 'JPN', 'MEA', 'MEX', 'ODA', 'SKO', 'UK', 'WEU', 'USA']
start_years = [2035]
lifetimes = [10, 15]
recycling_methods = ['pyrometallurgical', 'inorganic hydrometallurgical', 'direct recycling']
sizes = ['Large', 'Large SUV', 'Lower medium', 'Medium', 'Medium SUV', 'Mini', 'Small']
powertrains = ['battery electric','diesel hybrid','fuel cell electric','plugin diesel hybrid']
method = [m for m in bd.methods if 'IPCC 2021' in str(m) and 'climate change' in str(m) and 'GWP 100a, incl. H and bio CO2' in str(m)][0]
db_data = list(bd.Database(f'LCI_foreground_RCP60_2025'))
mileage_ranges = [(100000, 300000)]

# Number of iterations
num_iterations = 4000

# Run the simulation
results_df = monte_carlo_simulation(scenarios, regions, start_years, lifetimes, recycling_methods, sizes, powertrains, method, db_data, mileage_ranges, num_iterations=num_iterations, save_to_excel=True)
```

### Plot¶

In [39]:

```
# Find all files that match the pattern
file_pattern = 'MC_Final_20240903_081705*.xlsx'
file_list = glob.glob(file_pattern)

# Read and combine all files into a single DataFrame
dataframes = []
for file in file_list:
    df = pd.read_excel(file, sheet_name='Sheet1')
    dataframes.append(df)

# Concatenate all dataframes into one
combined_df = pd.concat(dataframes, ignore_index=True)

# Filter the data based on the allowed scenarios
allowed_scenarios = ['No Scenario','RCP60','RCP45','RCP26','RCP19']
combined_df = combined_df[combined_df['Scenario'].isin(allowed_scenarios)]

# Create a pivot table
pivot_df = combined_df.pivot_table(
    index=['Scenario', 'Region', 'Method', 'Total Mileage', 'Year', 'Type'],
    columns='Vehicle',
    values='Cumulative Value',
    aggfunc='sum'
).reset_index()

# Normalize BEV, FCEV, HEV, and PHEV values by dividing by Total Mileage and multiplying by 1000
vehicles_to_normalize = ['BEV', 'FCEV', 'HEV', 'PHEV']
for vehicle in vehicles_to_normalize:
    pivot_df[vehicle] = (pivot_df[vehicle] / pivot_df['Total Mileage']) * 1000

# Define the vehicles to compare and their colors
vehicles = ['BEV', 'HEV', 'PHEV', 'FCEV']
scenario_colors = {'No Scenario': '#696969', 'RCP60': 'tab:red', 'RCP45': 'tab:orange', 'RCP26': 'tab:blue', 'RCP19': 'tab:green'}
legend_labels = {'No Scenario': 'No Scenario', 'RCP60': 'SSP2-RCP6.0', 'RCP45': 'SSP2-RCP4.5', 'RCP26': 'SSP2-RCP2.6', 'RCP19': 'SSP2-RCP1.9'}

# Define the comparisons for the scatter plot
comparisons = [
    ('BEV', 'HEV'),
    ('BEV', 'PHEV'),
    ('BEV', 'FCEV'),
    ('PHEV', 'HEV'),
    ('PHEV', 'FCEV'),
    ('FCEV', 'HEV')
]

# Function to create scatter plot
def create_scatter_plot(pivot_df, limit_scatter_points=False):
    fig, axs = plt.subplots(2, 3, figsize=(10, 6))

    # Flatten axs array for easier indexing
    axs = axs.flatten()

    scatter_points_count = 0  # Counter for scatter points

    # Define different marker shapes for central points
    central_point_markers = ['o', 's', '^', 'D', 'P']  # Circle, square, triangle, diamond, pentagon

    # Define light colors for shading each vehicle type
    shading_colors = {
        'BEV': 'tab:green',
        'PHEV': 'tab:blue',
        'HEV': 'tab:orange',
        'FCEV': 'tab:pink'
    }

    # Loop over each comparison
    for idx, (vehicle1, vehicle2) in enumerate(comparisons):
        # Determine the subplot to use
        ax = axs[idx]

        # Set equal aspect ratio to ensure square plots
        ax.set_aspect('equal')

        # Filter data for the current subplot
        subplot_data = pivot_df[(pivot_df[vehicle1].notna()) & (pivot_df[vehicle2].notna())]

        if limit_scatter_points and len(subplot_data) > 3000:
            subplot_data = subplot_data.sample(n=3000, random_state=1)

        # Add shading: area above and below the diagonal
        ax.fill_between([0, 600], 0, [0, 600], color=shading_colors[vehicle1], alpha=0.15)
        ax.fill_between([0, 600], [0, 600], 600, color=shading_colors[vehicle2], alpha=0.15)

        # Create scatter plot with colors based on scenarios
        for i, scenario in enumerate(allowed_scenarios):
            scenario_data = subplot_data[subplot_data['Scenario'] == scenario]
            ax.scatter(scenario_data[vehicle1], scenario_data[vehicle2], marker='.', color=scenario_colors[scenario],
                       linewidth=0.5, alpha=0.7, s=15, label=legend_labels[scenario] if scatter_points_count == 0 else "")

            # Calculate mean values for the current scenario
            mean_x = scenario_data[vehicle1].mean()
            mean_y = scenario_data[vehicle2].mean()

            # Plot the mean values with different shapes
            ax.scatter(mean_x, mean_y, marker=central_point_markers[i % len(central_point_markers)], 
                       color=scenario_colors[scenario], edgecolor='black', s=50, zorder=5)

        # Increment scatter points count
        scatter_points_count += len(subplot_data)

        # Plot the diagonal line
        ax.plot([0, 600], [0, 600], color='black', linestyle='-', linewidth=0.5)

        # Set axis limits
        ax.set_xlim(0, 600)
        ax.set_ylim(0, 600)

        # Add labels
        ax.set_xlabel(f'{vehicle1} g CO2e per km')
        ax.set_ylabel(f'{vehicle2} g CO2e per km')

        # Add grid
        ax.grid(False)

    # Create a legend for the scatter plot colors
    handles, labels = axs[0].get_legend_handles_labels()
    #fig.legend(handles, labels, loc='upper center', bbox_to_anchor=(0.5, 1.05), ncol=4, frameon=False)

    # Adjust layout to make the plots square and fit well
    plt.tight_layout()

    # Save plot
    if limit_scatter_points:
        plt.savefig('MC_colored_scenarios_limited_square_shaded.svg', format='svg', transparent=True, bbox_inches='tight')
    else:
        plt.savefig('MC_colored_scenarios_square_shaded.svg', format='svg', transparent=True, bbox_inches='tight')

    # Show the plot
    plt.show()

    # Print scatter points count
    print("Total Scatter Points:", scatter_points_count)

# Create and show the scatter plot with square plots, different shapes for central points, and light shading
create_scatter_plot(pivot_df, limit_scatter_points=True)
```

```
Total Scatter Points: 18000
```

#### Calculate % differences¶

In [40]:

```
import pandas as pd
import numpy as np

def compare_vehicles(pivot_df, vehicle1, vehicle2):
    comparison_df = pivot_df[(pivot_df[vehicle1].notna()) & (pivot_df[vehicle2].notna())]

    # Determine wins
    comparison_df['Winner'] = np.where(comparison_df[vehicle1] < comparison_df[vehicle2], vehicle1, vehicle2)

    # Calculate statistics
    total_points = len(comparison_df)
    vehicle1_wins = (comparison_df['Winner'] == vehicle1).sum()
    vehicle2_wins = (comparison_df['Winner'] == vehicle2).sum()

    vehicle1_win_percentage = (vehicle1_wins / total_points) * 100
    vehicle2_win_percentage = (vehicle2_wins / total_points) * 100

    average_difference = (comparison_df[vehicle1] - comparison_df[vehicle2]).abs().mean()
    average_difference_percentage = (average_difference / comparison_df[[vehicle1, vehicle2]].mean().mean()) * 100

    # Calculate min, max, and mean values for each vehicle
    vehicle1_min = comparison_df[vehicle1].min()
    vehicle1_max = comparison_df[vehicle1].max()
    vehicle1_mean = comparison_df[vehicle1].mean()

    vehicle2_min = comparison_df[vehicle2].min()
    vehicle2_max = comparison_df[vehicle2].max()
    vehicle2_mean = comparison_df[vehicle2].mean()

    # Print table
    print(f"Comparison: {vehicle1} vs {vehicle2}")
    print(f"{vehicle1} wins: {vehicle1_wins} ({vehicle1_win_percentage:.2f}%)")
    print(f"{vehicle2} wins: {vehicle2_wins} ({vehicle2_win_percentage:.2f}%)")
    print(f"Average % difference: {average_difference_percentage:.2f}%")
    print(f"Total points compared: {total_points}")
    print(f"{vehicle1} - Min: {vehicle1_min:.2f}, Max: {vehicle1_max:.2f}, Mean: {vehicle1_mean:.2f}")
    print(f"{vehicle2} - Min: {vehicle2_min:.2f}, Max: {vehicle2_max:.2f}, Mean: {vehicle2_mean:.2f}\n")

# List of comparisons
comparisons = [
    ('BEV', 'HEV'),
    ('BEV', 'PHEV'),
    ('BEV', 'FCEV'),
    ('PHEV', 'HEV'),
    ('PHEV', 'FCEV'),
    ('FCEV', 'HEV')
]

# Compare each pair of vehicles
for vehicle1, vehicle2 in comparisons:
    compare_vehicles(pivot_df, vehicle1, vehicle2)
```

```
Comparison: BEV vs HEV
BEV wins: 2989 (99.63%)
HEV wins: 11 (0.37%)
Average % difference: 42.71%
Total points compared: 3000
BEV - Min: 48.38, Max: 487.50, Mean: 183.67
HEV - Min: 151.31, Max: 505.99, Mean: 283.28

Comparison: BEV vs PHEV
BEV wins: 2985 (99.50%)
PHEV wins: 15 (0.50%)
Average % difference: 24.43%
Total points compared: 3000
BEV - Min: 48.38, Max: 487.50, Mean: 183.67
PHEV - Min: 116.09, Max: 463.31, Mean: 234.69

Comparison: BEV vs FCEV
BEV wins: 2212 (73.73%)
FCEV wins: 788 (26.27%)
Average % difference: 30.44%
Total points compared: 3000
BEV - Min: 48.38, Max: 487.50, Mean: 183.67
FCEV - Min: -64.26, Max: 587.15, Mean: 197.05

Comparison: PHEV vs HEV
PHEV wins: 2959 (98.63%)
HEV wins: 41 (1.37%)
Average % difference: 18.80%
Total points compared: 3000
PHEV - Min: 116.09, Max: 463.31, Mean: 234.69
HEV - Min: 151.31, Max: 505.99, Mean: 283.28

Comparison: PHEV vs FCEV
PHEV wins: 929 (30.97%)
FCEV wins: 2071 (69.03%)
Average % difference: 25.30%
Total points compared: 3000
PHEV - Min: 116.09, Max: 463.31, Mean: 234.69
FCEV - Min: -64.26, Max: 587.15, Mean: 197.05

Comparison: FCEV vs HEV
FCEV wins: 2696 (89.87%)
HEV wins: 304 (10.13%)
Average % difference: 38.36%
Total points compared: 3000
FCEV - Min: -64.26, Max: 587.15, Mean: 197.05
HEV - Min: 151.31, Max: 505.99, Mean: 283.28
```

# 2 Running BEV vs. HEV differences¶

In [ ]:

```
start_time = time.time()
warnings.simplefilter(action='ignore', category=FutureWarning)

# Scenario parameters
scenarios = ['No Scenario','RCP26']
regions = ['AFR', 'AUS', 'CAN', 'CHI', 'CSA', 'EEU', 'FSU', 'IND', 'JPN', 'MEA', 'MEX', 'ODA', 'SKO', 'UK', 'WEU', 'USA']
sizes = ['Medium SUV']
start_years = [2025]
lifetimes = [15]
bounds = [200000]
recycling_methods = ['hydrometallurgical']

methods = []
methods.append([m for m in bd.methods if 'IPCC 2021' in str(m) and 'climate change' in str(m) and 'GWP 100a, incl. H and bio CO2' in str(m)][0])

# Store results in a dictionary
results_dict = {}

# Calculate total number of iterations for progress bar
total_iterations = len(regions) * len(recycling_methods) * len(start_years) * len(sizes) * len(scenarios) * len(lifetimes) * len(methods) * len(bounds)

# Use the fetched data
with tqdm(total=total_iterations, desc="Processing") as pbar:
    for region in regions:
        for recycling in recycling_methods:
            for years in start_years:
                nearest_year = nearest_available_year(years)
                for size in sizes:
                    for scenario in scenarios:
                        vehicles = []
                        try:
                            vehicles.append([x for x in bd.Database(f'LCI_foreground_RCP60_2025')
                                             if "transport, car, diesel hybrid," in x['name'] and 
                                             size in x['name'] and
                                             recycling in x['name'] and 
                                             str(nearest_year) in x['name'] and 
                                             region in x['location']][0])
                        except IndexError:
                            print(f"Diesel hybrid vehicle not found for year {nearest_year}, size {size}, region {region}, recycling {recycling}")
                        try:
                            vehicles.append([x for x in bd.Database(f'LCI_foreground_RCP60_2025')
                                             if "transport, car, battery electric," in x['name'] and 
                                             size in x['name'] and
                                             recycling in x['name'] and 
                                             str(nearest_year) in x['name'] and 
                                             region in x['location']][0])
                        except IndexError:
                            print(f"Battery electric vehicle not found for year {nearest_year}, size {size}, region {region}, recycling {recycling}")
                        
                        for vehicle in vehicles:
                            for lifetime in lifetimes:
                                for method in methods:
                                    for bound in bounds:
                                        df_vehicle_scenario = compute_cumulative(
                                            PLCA(vehicle, size, lifetime, method, scenario, region, start_year=years, end_year=(years+lifetime), system_expansion=(years+lifetime+1), step=5, lifecycle=200000, case=bound, location=region),
                                            years
                                        )
                                        df_vehicle_scenario['Recycling Method'] = recycling  # Add recycling method
                                        results_dict[(vehicle, size, lifetime, recycling, method, scenario, years, bound, region)] = df_vehicle_scenario
                                        pbar.update(1)  # Update progress bar after each iteration

results = pd.concat(results_dict.values(), ignore_index=True)
df_results = results
df_results.loc[df_results['Vehicle'].str.contains('transport, car, battery electric,', case=True, na=False), 'Vehicle'] = 'BEV'
df_results.loc[df_results['Vehicle'].str.contains('transport, car, diesel hybrid,', case=True, na=False), 'Vehicle'] = 'HEV'
end_time = time.time()
elapsed_time = (end_time - start_time) / 60
print(f"Execution time: {elapsed_time} minutes")
df_results.to_excel("pLCA_results_BEV_HEV.xlsx")
df_results
```

In [41]:

```
import pandas as pd
import matplotlib.pyplot as plt

# Load the Excel file into a DataFrame
df = pd.read_excel('Fig7_BEV-HEV-margins.xlsx')

# Processing the DataFrame
df = df.drop(columns=["Method", "Size", "Year", "Recycling Method"])
df = df[~df["Type"].isin(["Use", "Production"])]
df["Cumulative Value"] = df["Cumulative Value"] / df["Total Mileage"]

# Function to calculate percentage difference
def calculate_percentage_difference(df, scenario):
    filtered_df = df[df["Scenario"] == scenario]
    grouped = filtered_df.groupby(["Region", "Vehicle"])["Cumulative Value"].mean().unstack()
    bev_values = grouped["BEV"]
    hev_values = grouped["HEV"]
    percentage_difference = ((bev_values - hev_values) / hev_values) * 100
    return percentage_difference

# Calculate percentage differences for "No Scenario" and "RCP26"
percentage_difference_no_scenario = calculate_percentage_difference(df, "No Scenario")
percentage_difference_rcp26 = calculate_percentage_difference(df, "RCP26")

# Create a new DataFrame to hold the percentage differences
percentage_diff_df = pd.DataFrame({
    'Region': percentage_difference_no_scenario.index,
    'Percentage Difference (No Scenario)': percentage_difference_no_scenario.values,
    'Percentage Difference (RCP26)': percentage_difference_rcp26.values
})

# Export the DataFrame to an Excel file
output_file = 'percentage_differences.xlsx'
percentage_diff_df.to_excel(output_file, index=False)

# Prepare data for plotting
regions = percentage_difference_no_scenario.index
x = range(len(regions))
width = 0.35

# Define colors for the bars
color_no_scenario = '#843033'
color_rcp26 = '#51A5B2'

fig, ax = plt.subplots(figsize=(7, 3))

# Plotting the data
bars1 = ax.bar(x, percentage_difference_no_scenario, width, label='2025, LCA', color=color_no_scenario)
bars2 = ax.bar([p + width for p in x], percentage_difference_rcp26, width, label='2025, pLCA', color=color_rcp26)

# Adding labels and axis adjustments
ax.set_ylabel('%')
ax.set_title('Percentage GWP Margin between BEV and HEV')
ax.set_xticks([p + width / 2 for p in x])
ax.set_xticklabels(regions, rotation=90)
plt.ylim(-50, 0)

# Add a horizontal line at y=0
#ax.axhline(y=0, color='black', linewidth=0.5)

# Remove the legend
# ax.legend()  # Removed to eliminate the legend

plt.tight_layout()
plt.savefig("Margins.svg")
plt.show()

print(f"Data has been successfully saved to {output_file}")
```

```
Data has been successfully saved to percentage_differences.xlsx
```

# 3 Carbon footprint calculations for electricity, diesel, and hydrogen¶

In [ ]:

```
scenarios = ['RCP26']
regions = ['AFR', 'AUS', 'CAN', 'CHI', 'CSA', 'EEU', 'FSU', 'IND', 'JPN', 'MEA', 'MEX', 'ODA', 'SKO', 'UK', 'WEU', 'USA']
activities = ['market for hydrogen, gaseous','market for diesel, low-sulfur','market group for electricity, low voltage']
years = [2025, 2030, 2035, 2040, 2045, 2050]

# Initialize an empty DataFrame to store results
results_df = pd.DataFrame(columns=['Scenario', 'Region', 'Activity', 'Year', 'LCA Score'])

# Initialize the progress bar
total_iterations = len(activities) * len(scenarios) * len(years) * len(regions)
pbar = tqdm(total=total_iterations)

# Perform the LCA calculations
for activity in activities:
    for scenario in scenarios:
        for year in years:
            for region in regions:
                # Fetch all databases
                databases = bd.databases

                # Find the matching database
                db_name_prefix = f'ecoinvent_cutoff_3.9_tiam-ucl_SSP2-{scenario}_{year}'
                matching_db = next((db for db in databases if db.startswith(db_name_prefix)), None)

                if matching_db:
                    try:
                        x = [y for y in bd.Database(matching_db) if activity in y['name'] and region in y['location'] and "period" not in y['name']][0]
                        # Perform LCA
                        functional_unit = {x: 1}
                        method = [m for m in bd.methods if 'IPCC 2021' in str(m) and 'climate change' in str(m) and 'GWP 100a, incl. H and bio CO2' in str(m)][0]
                        lca = bc.LCA(functional_unit, method)
                        lca.lci()
                        lca.lcia()
                        score = lca.score
                        # Store the result in the DataFrame
                        new_row = pd.DataFrame([[scenario, region, activity, year, score]], columns=['Scenario', 'Region', 'Activity', 'Year', 'LCA Score'])
                        results_df = pd.concat([results_df, new_row], ignore_index=True)
                    except IndexError:
                        print(f"No activity found for {activity} in {region} for the year {year} under {scenario}")
                    except Exception as e:
                        print(f"Error calculating LCA for {activity} in {region} for the year {year} under {scenario}: {e}")
                else:
                    print(f"No matching database found for {db_name_prefix}")

                # Update the progress bar
                pbar.update(1)

# Close the progress bar
pbar.close()

# Save the results to an Excel file
results_df.to_excel('fuel_carbon_footprints.xlsx', index=False)

print("LCA calculation and results export completed.")
```

In [46]:

```
# Load the results from the provided Excel file
file_path = 'LCA_Results.xlsx'  # Change this to the path where you saved the file
results_df = pd.read_excel(file_path)

# Display the first few rows of the dataframe to understand its structure
print(results_df.head())

# Filter the DataFrame for the RCP26 scenario
rcp26_df = results_df[results_df['Scenario'] == 'RCP26']

# Function to calculate average carbon footprint per region, activity, and year
def calculate_average_carbon_footprint(df):
    avg_carbon_footprint_list = []

    for region in df['Region'].unique():
        for activity in df['Activity'].unique():
            # Get the baseline score for the year 2025 for the given region and activity
            baseline_score = df[(df['Year'] == 2025) & 
                                (df['Region'] == region) & 
                                (df['Activity'] == activity)]['LCA Score'].values[0]
            
            # Only consider the years 2025, 2030, 2035, and 2040
            scores = df[(df['Year'].isin([2025, 2030, 2035, 2040])) & 
                        (df['Region'] == region) & 
                        (df['Activity'] == activity)]['LCA Score'].values
            
            if len(scores) == 4:  # Ensure we have scores for all four years
                avg_score = np.mean(scores)
                avg_carbon_footprint_list.append({
                    'Scenario': 'RCP26',
                    'Region': region,
                    'Activity': activity,
                    'Avg Carbon Footprint': avg_score,
                    'Baseline Carbon Footprint': baseline_score
                })
    
    return pd.DataFrame(avg_carbon_footprint_list)

# Calculate average carbon footprints for RCP26
average_df = calculate_average_carbon_footprint(rcp26_df)
average_df.to_excel("1A.xlsx")

# Function to plot the average carbon footprints with baseline values and standard deviation
def plot_average_carbon_footprint(df, activity, colormap='viridis'):
    activity_df = df[df['Activity'] == activity]
    
    summary_df = activity_df.groupby(['Region']).agg(
        Avg_Carbon_Footprint=('Avg Carbon Footprint', 'mean'),
        Std_Carbon_Footprint=('Avg Carbon Footprint', 'std'),
        Baseline_Carbon_Footprint=('Baseline Carbon Footprint', 'mean')
    ).reset_index()

    # Order regions alphabetically
    summary_df = summary_df.sort_values('Region')

    # Create a normalizer
    norm = Normalize(vmin=summary_df['Avg_Carbon_Footprint'].min(), vmax=summary_df['Avg_Carbon_Footprint'].max())
    
    fig, ax = plt.subplots(figsize=(9, 3))
    x = np.arange(len(summary_df['Region']))
    width = 0.35

    # Define colors
    baseline_color = '#843033'
    avg_color = '#51A5B2'

    bars1 = ax.bar(x - width/2, summary_df['Baseline_Carbon_Footprint'], width, color=baseline_color, label='2025')
    bars2 = ax.bar(x + width/2, summary_df['Avg_Carbon_Footprint'], width, yerr=summary_df['Std_Carbon_Footprint'], color=avg_color, capsize=5, label='2025-2040')

    # Add horizontal line at y=0
    ax.axhline(y=0, color='black', linestyle='-', linewidth=0.6)

    #ax.set_xlabel('Region')
    ax.set_ylabel('kg CO2e per kg')
    ax.set_title(f'Electricity')
    ax.set_xticks(x)
    ax.set_xticklabels(summary_df['Region'])
    plt.xticks(rotation=90)
    ax.legend()

    plt.tight_layout()
    plt.savefig("fuel_carbon_footprints.svg")
    plt.show()

# Plot for the specified activity with the new color scheme
plot_average_carbon_footprint(average_df, 'market group for electricity, low voltage', colormap='viridis')
#plot_average_carbon_footprint(average_df, 'market for diesel, low-sulfur', colormap='viridis')
#plot_average_carbon_footprint(average_df, 'market group for electricity, low voltage', colormap='viridis')
```

```
  Scenario Region                                   Activity  Year  LCA Score
0    RCP60    AFR  market group for electricity, low voltage  2025   0.547455
1    RCP45    AFR  market group for electricity, low voltage  2025   0.547445
2    RCP26    AFR  market group for electricity, low voltage  2025   0.547445
3    RCP19    AFR  market group for electricity, low voltage  2025   0.547445
4    RCP60    AUS  market group for electricity, low voltage  2025   0.500445
```

# 4 Mileage breakeven analysis¶

#### Run vehicle comparisons are different mileages, scenarios, and regions.¶

In [ ]:

```
start_time = time.time()
warnings.simplefilter(action='ignore', category=FutureWarning)

# Scenario parameters
scenarios = ['No Scenario','RCP60','RCP45','RCP26','RCP19']
regions = ['AFR', 'AUS', 'CAN', 'CHI', 'CSA', 'EEU', 'FSU', 'IND', 'JPN', 'MEA', 'MEX', 'ODA', 'SKO', 'UK', 'WEU', 'USA']
sizes = ['Medium SUV']
start_years = [2025]
lifetimes = [10,20]
bounds = [50000,100000, 150000, 200000, 250000, 300000]
recycling_methods = ['pyrometallurgical']

methods = []
methods.append([m for m in bd.methods if 'IPCC 2021' in str(m) and 'climate change' in str(m) and 'GWP 100a, incl. H and bio CO2' in str(m)][0])

# Store results in a dictionary
results_dict = {}

# Calculate total number of iterations for progress bar
total_iterations = len(regions) * len(recycling_methods) * len(start_years) * len(sizes) * len(scenarios) * len(lifetimes) * len(methods) * len(bounds)

# Use the fetched data
with tqdm(total=total_iterations, desc="Processing") as pbar:
    for region in regions:
        for recycling in recycling_methods:
            for years in start_years:
                nearest_year = nearest_available_year(years)
                for size in sizes:
                    for scenario in scenarios:
                        vehicles = []
                        try:
                            vehicles.append([x for x in bd.Database(f'LCI_foreground_RCP60_2025')
                                             if "transport, car, diesel hybrid," in x['name'] and 
                                             size in x['name'] and
                                             recycling in x['name'] and 
                                             str(nearest_year) in x['name'] and 
                                             region in x['location']][0])
                        except IndexError:
                            print(f"Diesel hybrid vehicle not found for year {nearest_year}, size {size}, region {region}, recycling {recycling}")
                        try:
                            vehicles.append([x for x in bd.Database(f'LCI_foreground_RCP60_2025')
                                             if "transport, car, battery electric," in x['name'] and 
                                             size in x['name'] and
                                             recycling in x['name'] and 
                                             str(nearest_year) in x['name'] and 
                                             region in x['location']][0])
                        except IndexError:
                            print(f"Battery electric vehicle not found for year {nearest_year}, size {size}, region {region}, recycling {recycling}")
                        
                        for vehicle in vehicles:
                            for lifetime in lifetimes:
                                for method in methods:
                                    for bound in bounds:
                                        df_vehicle_scenario = compute_cumulative(
                                            PLCA(vehicle, size, lifetime, method, scenario, region, start_year=years, end_year=(years+lifetime), system_expansion=(years+lifetime+1), step=5, lifecycle=200000, case=bound, location=region),
                                            years
                                        )
                                        df_vehicle_scenario['Recycling Method'] = recycling  # Add recycling method
                                        results_dict[(vehicle, size, lifetime, recycling, method, scenario, years, bound, region)] = df_vehicle_scenario
                                        pbar.update(1)  # Update progress bar after each iteration

results = pd.concat(results_dict.values(), ignore_index=True)
df_results = results
df_results.loc[df_results['Vehicle'].str.contains('transport, car, battery electric,', case=True, na=False), 'Vehicle'] = 'BEV'
df_results.loc[df_results['Vehicle'].str.contains('transport, car, diesel hybrid,', case=True, na=False), 'Vehicle'] = 'HEV'
end_time = time.time()
elapsed_time = (end_time - start_time) / 60
print(f"Execution time: {elapsed_time} minutes")
df_results.to_excel("Fig8_carbon_breakeven_10s_20s_lifetimes.xlsx")
df_results
```

In [ ]:

```
# Suppress warnings
warnings.filterwarnings('ignore')

# Load the data
file_path = 'LCA_results.xlsx'
df = pd.read_excel(file_path)
#df_results = pd.read_excel("Carbon intensity breakever.xlsx")
df_results = pd.read_excel("Fig8_all.xlsx")

# Filter out rows with 'market for diesel, low-sulfur' in the 'Activity' column
df = df[df['Activity'] != 'market for diesel, low-sulfur']
df = df[df['Activity'] != 'market for hydrogen, gaseous']

# Rename scenarios in 2025 to 'Baseline'
df.loc[df['Year'] == 2025, 'Scenario'] = 'Baseline'

# Drop duplicates for 2025 'Baseline' scenario
df = df.drop_duplicates(subset=['Region', 'Activity', 'Year', 'Scenario'])

# Function to compute average LCA Score
def compute_average_lca(df, base_year, end_year):
    baseline_lca = df[(df['Year'] == base_year) & (df['Scenario'] == 'Baseline')]['LCA Score'].values[0]
    target_lca = df[df['Year'] == end_year]['LCA Score'].values
    return (baseline_lca + target_lca) / 2

# Compute the average LCA scores for the specified year ranges
years = [2030, 2035, 2040]
results = []

for region in df['Region'].unique():
    region_df = df[df['Region'] == region]
    baseline_year = 2025
    for year in years:
        temp_df = region_df[(region_df['Year'] == year) & (region_df['Scenario'] != 'Baseline')]
        avg_lca_scores = compute_average_lca(region_df, baseline_year, year)
        temp_df['LCA Score'] = avg_lca_scores
        temp_df['Lifetime'] = year - baseline_year
        results.append(temp_df)

# Combine results into a single DataFrame
final_df = pd.concat(results).sort_values(by=['Region', 'Scenario', 'Year']).reset_index(drop=True)

# Add the Baseline rows
baseline_rows = df[(df['Year'] == 2025) & (df['Scenario'] == 'Baseline')]
baseline_rows['Lifetime'] = 15

# Concatenate the baseline rows with the final dataframe
final_df = pd.concat([baseline_rows, final_df]).sort_values(by=['Region', 'Scenario', 'Year']).reset_index(drop=True)
final_df
```

In [ ]:

```
import pandas as pd
import warnings

# Suppress warnings
warnings.filterwarnings('ignore')

# Load the data
file_path = 'LCA_results.xlsx'
df = pd.read_excel(file_path)
#df_results = pd.read_excel("Carbon intensity breakever.xlsx")
df_results = pd.read_excel("Fig8_all.xlsx")

# Filter out rows with 'market for diesel, low-sulfur' in the 'Activity' column
df = df[df['Activity'] != 'market for diesel, low-sulfur']
df = df[df['Activity'] != 'market for hydrogen, gaseous']

# Rename scenarios in 2025 to 'Baseline'
df.loc[df['Year'] == 2025, 'Scenario'] = 'Baseline'

# Drop duplicates for 2025 'Baseline' scenario
df = df.drop_duplicates(subset=['Region', 'Activity', 'Year', 'Scenario'])

# Function to compute average LCA Score
def compute_average_lca(df, base_year, end_year):
    baseline_lca = df[(df['Year'] == base_year) & (df['Scenario'] == 'Baseline')]['LCA Score'].values[0]
    target_lca = df[df['Year'] == end_year]['LCA Score'].values
    return (baseline_lca + target_lca) / 2

# Compute the average LCA scores for the specified year ranges
years = [2030, 2035, 2040, 2045]
results = []

for region in df['Region'].unique():
    region_df = df[df['Region'] == region]
    baseline_year = 2025
    for year in years:
        temp_df = region_df[(region_df['Year'] == year) & (region_df['Scenario'] != 'Baseline')]
        avg_lca_scores = compute_average_lca(region_df, baseline_year, year)
        temp_df['LCA Score'] = avg_lca_scores
        temp_df['Lifetime'] = year - baseline_year
        results.append(temp_df)

# Combine results into a single DataFrame
final_df = pd.concat(results).sort_values(by=['Region', 'Scenario', 'Year']).reset_index(drop=True)

# Add the Baseline rows
baseline_rows = df[(df['Year'] == 2025) & (df['Scenario'] == 'Baseline')]
baseline_rows['Lifetime'] = 15

# Concatenate the baseline rows with the final dataframe
final_df = pd.concat([baseline_rows, final_df]).sort_values(by=['Region', 'Scenario', 'Year']).reset_index(drop=True)
final_df = final_df[final_df['Lifetime'].isin([0, 15])]

# Filter df_results to only include 'End-of-life' rows
df_results_filtered = df_results[df_results['Type'] == 'End-of-life'].copy()

# Create a dictionary to map (Scenario, Lifetime, Region) to LCA Score
lca_score_mapping = final_df.set_index(['Scenario', 'Lifetime', 'Region'])['LCA Score'].to_dict()

# Function to get the LCA score based on Scenario, Lifetime, and Region
def get_lca_score(row):
    key = (row['Scenario'], row['Lifetime'], row['Region'])
    return lca_score_mapping.get(key, None)  # None if not found

# Add the 'Average carbon intensity' column to the filtered dataframe
df_results_filtered['Average carbon intensity'] = df_results_filtered.apply(get_lca_score, axis=1)

# Optional: If you want to merge this back to the original df_results
df_results.update(df_results_filtered)

# Save the updated dataframe to a new Excel file if needed
df_results_filtered.to_excel('df_results_updated.xlsx', index=False)
df_results_filtered
```

In [ ]:

```
import pandas as pd
import warnings

# Suppress warnings
warnings.filterwarnings('ignore')

# Load the data
file_path = 'LCA_results.xlsx'
df = pd.read_excel(file_path)
#df_results = pd.read_excel("Carbon intensity breakever.xlsx")
df_results = pd.read_excel("Fig8_all.xlsx")

# Filter out rows with 'market for diesel, low-sulfur' in the 'Activity' column
df = df[df['Activity'] != 'market for diesel, low-sulfur']
df = df[df['Activity'] != 'market for hydrogen, gaseous']

# Rename scenarios in 2025 to 'Baseline'
df.loc[df['Year'] == 2025, 'Scenario'] = 'Baseline'

# Drop duplicates for 2025 'Baseline' scenario
df = df.drop_duplicates(subset=['Region', 'Activity', 'Year', 'Scenario'])

# Function to compute average LCA Score
def compute_average_lca(df, base_year, end_year):
    baseline_lca = df[(df['Year'] == base_year) & (df['Scenario'] == 'Baseline')]['LCA Score'].values[0]
    target_lca = df[df['Year'] == end_year]['LCA Score'].values
    return (baseline_lca + target_lca) / 2

# Compute the average LCA scores for the specified year ranges (including 2030 for 10-year lifetime)
years = [2030, 2035, 2040, 2045]  # Adding 2030 for 10-year lifetime
results = []

for region in df['Region'].unique():
    region_df = df[df['Region'] == region]
    baseline_year = 2025
    for year in years:
        temp_df = region_df[(region_df['Year'] == year) & (region_df['Scenario'] != 'Baseline')]
        avg_lca_scores = compute_average_lca(region_df, baseline_year, year)
        temp_df['LCA Score'] = avg_lca_scores
        temp_df['Lifetime'] = year - baseline_year  # Lifetime is the difference between the target year and 2025
        results.append(temp_df)

# Combine results into a single DataFrame
final_df = pd.concat(results).sort_values(by=['Region', 'Scenario', 'Year']).reset_index(drop=True)

# Add the Baseline rows
baseline_rows = df[(df['Year'] == 2025) & (df['Scenario'] == 'Baseline')]

# Duplicate the Baseline rows for 10, 15, and 20 years of lifetime
for lifetime in [10, 15, 20]:
    temp_baseline = baseline_rows.copy()
    temp_baseline['Lifetime'] = lifetime  # Set lifetime to 10, 15, and 20
    final_df = pd.concat([final_df, temp_baseline])

# Sort and reset index
final_df = final_df.sort_values(by=['Region', 'Scenario', 'Year']).reset_index(drop=True)

# Filter final_df for Lifetime values of 10, 15, and 20 years
final_df = final_df[final_df['Lifetime'].isin([10, 15, 20])]

# Filter df_results to only include 'End-of-life' rows
df_results_filtered = df_results[df_results['Type'] == 'End-of-life'].copy()

# Create a dictionary to map (Scenario, Lifetime, Region) to LCA Score
lca_score_mapping = final_df.set_index(['Scenario', 'Lifetime', 'Region'])['LCA Score'].to_dict()

# Function to get the LCA score based on Scenario, Lifetime, and Region
def get_lca_score(row):
    key = (row['Scenario'], row['Lifetime'], row['Region'])
    return lca_score_mapping.get(key, None)  # Return None if the combination is not found

# Add the 'Average carbon intensity' column to the filtered dataframe
df_results_filtered['Average carbon intensity'] = df_results_filtered.apply(get_lca_score, axis=1)

# Optional: If you want to merge this back to the original df_results
df_results.update(df_results_filtered)

# Save the updated dataframe to a new Excel file if needed
df_results_filtered.to_excel('df_results_updated_with_10_15_20_year_lifetimes.xlsx', index=False)

df_results_filtered
```

In [ ]:

```
import pandas as pd
import numpy as np

# Load the data
file_path = 'df_results_updated_with_10_15_20_year_lifetimes.xlsx'
df = pd.read_excel(file_path)

# Filter the required columns
df_filtered = df[['Scenario', 'Region', 'Vehicle', 'Total Mileage', 'Cumulative Value', 'Average carbon intensity']]

# Separate HEV and BEV data
df_hev = df_filtered[df_filtered['Vehicle'] == 'HEV']
df_bev = df_filtered[df_filtered['Vehicle'] == 'BEV']

# Initialize an empty list to store results
intersections = []

# Loop through each unique combination of Scenario and Region
for scenario in df_filtered['Scenario'].unique():
    for region in df_filtered['Region'].unique():
        # Filter data for the current Scenario and Region
        hev_data = df_hev[(df_hev['Scenario'] == scenario) & (df_hev['Region'] == region)]
        bev_data = df_bev[(df_bev['Scenario'] == scenario) & (df_bev['Region'] == region)]
        
        if hev_data.empty or bev_data.empty:
            continue

        # Interpolate to find the intersection point
        for i in range(1, len(hev_data)):
            hev_prev = hev_data.iloc[i-1]
            hev_curr = hev_data.iloc[i]
            
            for j in range(1, len(bev_data)):
                bev_prev = bev_data.iloc[j-1]
                bev_curr = bev_data.iloc[j]
                
                # Check if the lines intersect
                if (hev_prev['Cumulative Value'] <= bev_prev['Cumulative Value'] and hev_curr['Cumulative Value'] >= bev_curr['Cumulative Value']) or \
                   (hev_prev['Cumulative Value'] >= bev_prev['Cumulative Value'] and hev_curr['Cumulative Value'] <= bev_curr['Cumulative Value']):
                    # Linear interpolation to find the intersection mileage
                    hev_slope = (hev_curr['Cumulative Value'] - hev_prev['Cumulative Value']) / (hev_curr['Total Mileage'] - hev_prev['Total Mileage'])
                    bev_slope = (bev_curr['Cumulative Value'] - bev_prev['Cumulative Value']) / (bev_curr['Total Mileage'] - bev_prev['Total Mileage'])
                    
                    intersect_mileage = (bev_prev['Cumulative Value'] - hev_prev['Cumulative Value'] + hev_slope * hev_prev['Total Mileage'] - bev_slope * bev_prev['Total Mileage']) / (hev_slope - bev_slope)
                    
                    # Interpolating Average carbon intensity
                    hev_carbon_slope = (hev_curr['Average carbon intensity'] - hev_prev['Average carbon intensity']) / (hev_curr['Total Mileage'] - hev_prev['Total Mileage'])
                    bev_carbon_slope = (bev_curr['Average carbon intensity'] - bev_prev['Average carbon intensity']) / (bev_curr['Total Mileage'] - bev_prev['Total Mileage'])
                    
                    intersect_carbon_intensity = hev_prev['Average carbon intensity'] + hev_carbon_slope * (intersect_mileage - hev_prev['Total Mileage'])
                    
                    intersections.append({
                        'Scenario': scenario,
                        'Region': region,
                        'Intersection Mileage': intersect_mileage,
                        'Average carbon intensity': intersect_carbon_intensity
                    })

# Create a DataFrame from the results
intersection_df = pd.DataFrame(intersections)

# Save the result to an Excel file
intersection_df.to_excel('intersection_mileage.xlsx', index=False)

# Display the result
#intersection_df = intersection_df.drop_duplicates(subset=['Scenario', 'Region','Intersection Mileage','Average carbon intensity'])
intersection_df
```

In [ ]:

```
import pandas as pd
import numpy as np

# Load the data
file_path = 'df_results_updated_with_10_15_20_year_lifetimes.xlsx'
df = pd.read_excel(file_path)

# Filter the required columns including 'Lifetime'
df_filtered = df[['Scenario', 'Region', 'Vehicle', 'Lifetime', 'Total Mileage', 'Cumulative Value', 'Average carbon intensity']]

# Separate HEV and BEV data
df_hev = df_filtered[df_filtered['Vehicle'] == 'HEV']
df_bev = df_filtered[df_filtered['Vehicle'] == 'BEV']

# Initialize an empty list to store results
intersections = []

# Loop through each unique combination of Scenario, Region, and Lifetime
for scenario in df_filtered['Scenario'].unique():
    for region in df_filtered['Region'].unique():
        for lifetime in df_filtered['Lifetime'].unique():
            # Filter data for the current Scenario, Region, and Lifetime
            hev_data = df_hev[(df_hev['Scenario'] == scenario) & (df_hev['Region'] == region) & (df_hev['Lifetime'] == lifetime)]
            bev_data = df_bev[(df_bev['Scenario'] == scenario) & (df_bev['Region'] == region) & (df_bev['Lifetime'] == lifetime)]
            
            if hev_data.empty or bev_data.empty:
                continue

            # Interpolate to find the intersection point
            for i in range(1, len(hev_data)):
                hev_prev = hev_data.iloc[i-1]
                hev_curr = hev_data.iloc[i]
                
                for j in range(1, len(bev_data)):
                    bev_prev = bev_data.iloc[j-1]
                    bev_curr = bev_data.iloc[j]
                    
                    # Check if the lines intersect
                    if (hev_prev['Cumulative Value'] <= bev_prev['Cumulative Value'] and hev_curr['Cumulative Value'] >= bev_curr['Cumulative Value']) or \
                       (hev_prev['Cumulative Value'] >= bev_prev['Cumulative Value'] and hev_curr['Cumulative Value'] <= bev_curr['Cumulative Value']):
                        # Linear interpolation to find the intersection mileage
                        hev_slope = (hev_curr['Cumulative Value'] - hev_prev['Cumulative Value']) / (hev_curr['Total Mileage'] - hev_prev['Total Mileage'])
                        bev_slope = (bev_curr['Cumulative Value'] - bev_prev['Cumulative Value']) / (bev_curr['Total Mileage'] - bev_prev['Total Mileage'])
                        
                        intersect_mileage = (bev_prev['Cumulative Value'] - hev_prev['Cumulative Value'] + hev_slope * hev_prev['Total Mileage'] - bev_slope * bev_prev['Total Mileage']) / (hev_slope - bev_slope)
                        
                        # Interpolating Average carbon intensity
                        hev_carbon_slope = (hev_curr['Average carbon intensity'] - hev_prev['Average carbon intensity']) / (hev_curr['Total Mileage'] - hev_prev['Total Mileage'])
                        bev_carbon_slope = (bev_curr['Average carbon intensity'] - bev_prev['Average carbon intensity']) / (bev_curr['Total Mileage'] - bev_prev['Total Mileage'])
                        
                        intersect_carbon_intensity = hev_prev['Average carbon intensity'] + hev_carbon_slope * (intersect_mileage - hev_prev['Total Mileage'])
                        
                        intersections.append({
                            'Scenario': scenario,
                            'Region': region,
                            'Lifetime': lifetime,  # Include the lifetime information
                            'Intersection Mileage': intersect_mileage,
                            'Average carbon intensity': intersect_carbon_intensity
                        })

# Create a DataFrame from the results including the 'Lifetime' column
intersection_df = pd.DataFrame(intersections)

# Group by 'Scenario', 'Region', and 'Lifetime' and average 'Intersection Mileage' and 'Average carbon intensity'
intersection_avg_df = intersection_df.groupby(['Scenario', 'Region', 'Lifetime']).agg({
    'Intersection Mileage': 'mean',
    'Average carbon intensity': 'mean'
}).reset_index()

# Save the result to an Excel file
intersection_avg_df.to_excel('intersection_mileage_avg_lifetime.xlsx', index=False)
intersection_avg_df = intersection_avg_df.drop(columns=['Lifetime'])

# Display the result
intersection_avg_df
# Display the result
intersection_df = intersection_avg_df
intersection_df
```

In [43]:

```
import pandas as pd
import numpy as np

# Load the data
file_path = 'df_results_updated_with_10_15_20_year_lifetimes.xlsx'
df = pd.read_excel(file_path)

# Filter the required columns
df_filtered = df[['Scenario', 'Region', 'Vehicle', 'Total Mileage', 'Cumulative Value', 'Average carbon intensity']]

# Separate HEV and BEV data
df_hev = df_filtered[df_filtered['Vehicle'] == 'HEV']
df_bev = df_filtered[df_filtered['Vehicle'] == 'BEV']

# Initialize an empty list to store results
intersections = []

# Loop through each unique combination of Scenario and Region
for scenario in df_filtered['Scenario'].unique():
    for region in df_filtered['Region'].unique():
        # Filter data for the current Scenario and Region
        hev_data = df_hev[(df_hev['Scenario'] == scenario) & (df_hev['Region'] == region)]
        bev_data = df_bev[(df_bev['Scenario'] == scenario) & (df_bev['Region'] == region)]
        
        if hev_data.empty or bev_data.empty:
            continue

        # Interpolate to find the intersection point
        for i in range(1, len(hev_data)):
            hev_prev = hev_data.iloc[i-1]
            hev_curr = hev_data.iloc[i]
            
            for j in range(1, len(bev_data)):
                bev_prev = bev_data.iloc[j-1]
                bev_curr = bev_data.iloc[j]
                
                # Check if the lines intersect
                if (hev_prev['Cumulative Value'] <= bev_prev['Cumulative Value'] and hev_curr['Cumulative Value'] >= bev_curr['Cumulative Value']) or \
                   (hev_prev['Cumulative Value'] >= bev_prev['Cumulative Value'] and hev_curr['Cumulative Value'] <= bev_curr['Cumulative Value']):
                    # Linear interpolation to find the intersection mileage
                    hev_slope = (hev_curr['Cumulative Value'] - hev_prev['Cumulative Value']) / (hev_curr['Total Mileage'] - hev_prev['Total Mileage'])
                    bev_slope = (bev_curr['Cumulative Value'] - bev_prev['Cumulative Value']) / (bev_curr['Total Mileage'] - bev_prev['Total Mileage'])
                    
                    intersect_mileage = (bev_prev['Cumulative Value'] - hev_prev['Cumulative Value'] + hev_slope * hev_prev['Total Mileage'] - bev_slope * bev_prev['Total Mileage']) / (hev_slope - bev_slope)
                    
                    # Interpolating Average carbon intensity
                    hev_carbon_slope = (hev_curr['Average carbon intensity'] - hev_prev['Average carbon intensity']) / (hev_curr['Total Mileage'] - hev_prev['Total Mileage'])
                    bev_carbon_slope = (bev_curr['Average carbon intensity'] - bev_prev['Average carbon intensity']) / (bev_curr['Total Mileage'] - bev_prev['Total Mileage'])
                    
                    intersect_carbon_intensity = hev_prev['Average carbon intensity'] + hev_carbon_slope * (intersect_mileage - hev_prev['Total Mileage'])
                    
                    intersections.append({
                        'Scenario': scenario,
                        'Region': region,
                        'Intersection Mileage': intersect_mileage,
                        'Average carbon intensity': intersect_carbon_intensity
                    })

# Create a DataFrame from the results
intersection_df = pd.DataFrame(intersections)

# Group by 'Scenario' and 'Region' and average 'Intersection Mileage' and 'Average carbon intensity'
intersection_avg_df = intersection_df.groupby(['Scenario', 'Region']).agg({
    'Intersection Mileage': 'mean',
    'Average carbon intensity': 'mean'
}).reset_index()

# Save the result to an Excel file
intersection_avg_df.to_excel('intersection_mileage_avg.xlsx', index=False)

# Display the result
intersection_df = intersection_avg_df
intersection_df
```

Out[43]:

|  | Scenario | Region | Intersection Mileage | Average carbon intensity |
| --- | --- | --- | --- | --- |
| 0 | Baseline | AFR | 118099.121978 | 0.547455 |
| 1 | Baseline | AUS | 112283.194543 | 0.500445 |
| 2 | Baseline | CAN | 53859.875954 | 0.186894 |
| 3 | Baseline | CHI | 129823.580975 | 0.643863 |
| 4 | Baseline | CSA | 52183.952072 | 0.141840 |
| ... | ... | ... | ... | ... |
| 75 | RCP60 | ODA | 63688.063574 | 0.373086 |
| 76 | RCP60 | SKO | 110043.407481 | 0.492695 |
| 77 | RCP60 | UK | 41298.299667 | 0.232268 |
| 78 | RCP60 | USA | 77929.070764 | 0.357895 |
| 79 | RCP60 | WEU | 61526.731747 | 0.284378 |

80 rows × 4 columns

In [44]:

```
import numpy as np
import matplotlib.pyplot as plt
from scipy.optimize import curve_fit

# Define the rational function
def rational(x, a, b, c, d):
    return (a * x + b) / (c * x + d)

# Extract x, y data and region labels
x = intersection_df['Intersection Mileage']
y = intersection_df['Average carbon intensity']
regions = intersection_df['Region']
scenarios = intersection_df['Scenario']

# Provide initial guesses for the parameters
initial_guesses = [1, 1, 1, 1]

# Use curve_fit to find the best fit parameters with initial guesses
params, covariance = curve_fit(rational, x, y, p0=initial_guesses, maxfev=10000)
a, b, c, d = params

# Generate values for the fitted rational function, extending the range of x to cover the forecast
x_fit = np.linspace(x.min(), x.max(), 100)
y_fit = rational(x_fit, a, b, c, d)

# Extend the range of x to forecast carbon intensities up to 200000 km
x_forecast = np.linspace(x.min(), 220000, 100)
y_forecast = rational(x_forecast, a, b, c, d)

# Calculate the residuals and standard deviation
residuals = y - rational(x, a, b, c, d)
std_dev = np.std(residuals)

# Create upper and lower bounds
y_upper = y_forecast + std_dev
y_lower = y_forecast - std_dev

# Create a scatter plot with different markers and colors based on "Scenario"
plt.figure(figsize=(10, 4))

# Plot based on different scenarios with unique markers and colors
scenario_styles = {
    'Baseline': {'color': '#4d4d4d', 'marker': 'o', 'label': 'Baseline'},
    'RCP60': {'color': 'red', 'marker': 's', 'label': 'RCP60'},
    'RCP45': {'color': 'orange', 'marker': '^', 'label': 'RCP45'},
    'RCP26': {'color': 'blue', 'marker': 'D', 'label': 'RCP26'},
    'RCP19': {'color': 'green', 'marker': 'P', 'label': 'RCP19'}
}

for scenario, style in scenario_styles.items():
    mask = (scenarios == scenario)
    plt.scatter(x[mask], y[mask], c=style['color'], alpha=0.5, marker=style['marker'], s=30, label=style['label'])

# Plot the rational fit line
plt.plot(x_forecast, y_forecast, color='black', linewidth=1)

#843033

# Plot the standard deviation bounds
plt.fill_between(x_forecast, y_lower, y_upper, color='black', alpha=0.15)
#366F78 colour before

# Adding title and labels
plt.xlabel('Breakeven mileage - km')
plt.ylabel('Electricity carbon intensity - kg CO2e per kWh')

# Show the plot without legend
plt.grid(False)
plt.savefig("breakeven.svg")
plt.show()
```

# 5 Global sensitivity analysis¶

In [ ]:

```
warnings.filterwarnings('ignore')

# Existing Parameters:
scenarios = ['No Scenario', 'RCP60', 'RCP45', 'RCP26', 'RCP19']
regions = ['AFR', 'AUS', 'CAN', 'CHI', 'CSA', 'EEU', 'FSU', 'IND', 'JPN', 'MEA', 'MEX', 'ODA', 'SKO', 'UK', 'WEU', 'USA']
lifetimes = [10, 15, 20]
recycling_methods = ['pyrometallurgical', 'inorganic hydrometallurgical', 'direct recycling']
sizes = ['Large', 'Large SUV', 'Lower medium', 'Medium', 'Medium SUV', 'Mini', 'Small']
method = [m for m in bd.methods if 'IPCC 2021' in str(m) and 'climate change' in str(m) and 'GWP 100a, incl. H and bio CO2' in str(m)][0]

# Define the problem for GSA
problem_with_region = {
    'num_vars': 6,
    'names': ['Scenario', 'Region', 'Lifetime', 'Battery Recycling Method', 'Vehicle Size', 'Mileage'],
    'bounds': [[-0.5, 4.5], [-0.5, 15.5], [-0.5, 2.5], [-0.5, 2.5], [-0.5, 6.5], [100000, 300000]]
}

problem_without_region = {
    'num_vars': 5,
    'names': ['Scenario', 'Lifetime', 'Battery Recycling Method', 'Vehicle Size', 'Mileage'],
    'bounds': [[-0.5, 4.5], [-0.5, 2.5], [-0.5, 2.5], [-0.5, 6.5], [100000, 300000]]
}

# Generate samples for GSA
number_of_trajectories = 50  # Increase the number of trajectories for better results
sample_with_region = saltelli.sample(problem_with_region, number_of_trajectories, calc_second_order=True)
sample_without_region = saltelli.sample(problem_without_region, number_of_trajectories, calc_second_order=True)

print(f"Sample with region shape: {sample_with_region.shape}")
print(f"Sample without region shape: {sample_without_region.shape}")

def get_vehicle_data(vehicle_type, exact_size_pattern, selected_recycling_method, vehicle_year, selected_region):
    vehicles = []
    vehicle_map = {
        'BEV': 'transport, car, battery electric,',
        'HEV': 'transport, car, diesel hybrid,',
        'FCEV': 'transport, car, fuel cell electric,',
        'PHEV': 'transport, car, plugin diesel hybrid,'
    }
    vehicle_type_str = vehicle_map[vehicle_type]

    vehicle = next((x for x in bd.Database('LCI_foreground_RCP60_2025') if 
                    vehicle_type_str in x['name'] and
                    re.search(exact_size_pattern, x['name']) and
                    selected_recycling_method in x['name'] and
                    vehicle_year in x['name'] and
                    selected_region in x['location']), None)
    if vehicle:
        vehicles.append(vehicle)
    return vehicles

def compute_gsa_analysis(type_of_analysis, vehicle_type1, vehicle_type2=None, region=None):
    start_time = time.time()
    
    if type_of_analysis == "arbitrary_score":
        model = lambda inputs: arbitrary_score_gsa(inputs, vehicle_type1)
        filename_suffix = "Arb"
        sample = sample_with_region
    elif type_of_analysis == "outcome_vs_vehicle":
        model = lambda inputs: outcome_vs_vehicle_gsa(inputs, vehicle_type1, vehicle_type2)
        filename_suffix = f"OutcomeVs{vehicle_type2}"
        sample = sample_with_region
    elif type_of_analysis == "outcome_within_region":
        model = lambda inputs: outcome_within_region_gsa(inputs, vehicle_type1, vehicle_type2, region)
        filename_suffix = f"OutcomeWithinRegion_{region}"
        sample = sample_without_region
    else:
        raise ValueError("Invalid type of analysis selected")

    output = np.array([model(x) for x in tqdm(sample, desc="Processing samples", total=len(sample))])
    results = sobol.analyze(problem_with_region if type_of_analysis != "outcome_within_region" else problem_without_region, output, print_to_console=False)
    
    sobol_indices_df = pd.DataFrame({
        'Parameter': (problem_with_region if type_of_analysis != "outcome_within_region" else problem_without_region)['names'],
        'First_Order_Sobol_Index': results['S1'],
        'Total_Order_Sobol_Index': results['ST']
    })
    
    sobol_indices_df.loc[len(sobol_indices_df.index)] = ['Number of Trajectories', number_of_trajectories, '']
    timestamp = datetime.now().strftime("%Y%m%d_%H%M%S")
    excel_filename = f"GSA_{vehicle_type1}_{filename_suffix}_{timestamp}.xlsx"
    sobol_indices_df.to_excel(excel_filename, index=False)
    print(f"Sobol indices saved as {excel_filename}")

    end_time = time.time()
    elapsed_time = (end_time - start_time) / 60
    print(f"Elapsed time: {elapsed_time:.2f} minutes")

def arbitrary_score_gsa(inputs, vehicle_type):
    selected_scenario = scenarios[int(inputs[0])]
    selected_region = regions[int(inputs[1])]
    selected_lifetime = lifetimes[int(inputs[2])]
    selected_recycling_method = recycling_methods[int(inputs[3])]
    selected_size = sizes[int(inputs[4])]
    selected_mileages = int(inputs[5])
    selected_start_year = 2025
    vehicle_year = determine_vehicle_year(selected_start_year)
    exact_size_pattern = r'\b' + re.escape(selected_size) + r'\b'
    vehicles = get_vehicle_data(vehicle_type, exact_size_pattern, selected_recycling_method, vehicle_year, selected_region)
    
    results = compute_results(vehicles, selected_size, selected_lifetime, method, selected_scenario, selected_region, selected_start_year, selected_mileages)
    total_GWP = calculate_gwp(results, 'kg CO2 per km')
    return total_GWP

def outcome_vs_vehicle_gsa(inputs, vehicle_type1, vehicle_type2):
    selected_scenario = scenarios[int(inputs[0])]
    selected_region = regions[int(inputs[1])]
    selected_lifetime = lifetimes[int(inputs[2])]
    selected_recycling_method = recycling_methods[int(inputs[3])]
    selected_size = sizes[int(inputs[4])]
    selected_mileages = int(inputs[5])
    selected_start_year = 2025
    vehicle_year = determine_vehicle_year(selected_start_year)
    exact_size_pattern = r'\b' + re.escape(selected_size) + r'\b'
    
    vehicles1 = get_vehicle_data(vehicle_type1, exact_size_pattern, selected_recycling_method, vehicle_year, selected_region)
    vehicles2 = get_vehicle_data(vehicle_type2, exact_size_pattern, selected_recycling_method, vehicle_year, selected_region)
    
    results1 = compute_results(vehicles1, selected_size, selected_lifetime, method, selected_scenario, selected_region, selected_start_year, selected_mileages)
    results2 = compute_results(vehicles2, selected_size, selected_lifetime, method, selected_scenario, selected_region, selected_start_year, selected_mileages)
    
    total_GWP = calculate_winning_margin(results1, results2, 'kg CO2 per km')
    return total_GWP

def outcome_within_region_gsa(inputs, vehicle_type1, vehicle_type2, region):
    selected_scenario = scenarios[int(inputs[0])]
    selected_lifetime = lifetimes[int(inputs[1])]
    selected_recycling_method = recycling_methods[int(inputs[2])]
    selected_size = sizes[int(inputs[3])]
    selected_mileages = int(inputs[4])
    selected_start_year = 2025
    vehicle_year = determine_vehicle_year(selected_start_year)
    exact_size_pattern = r'\b' + re.escape(selected_size) + r'\b'
    
    vehicles1 = get_vehicle_data(vehicle_type1, exact_size_pattern, selected_recycling_method, vehicle_year, region)
    vehicles2 = get_vehicle_data(vehicle_type2, exact_size_pattern, selected_recycling_method, vehicle_year, region)
    
    results1 = compute_results(vehicles1, selected_size, selected_lifetime, method, selected_scenario, region, selected_start_year, selected_mileages)
    results2 = compute_results(vehicles2, selected_size, selected_lifetime, method, selected_scenario, region, selected_start_year, selected_mileages)
    
    total_GWP = calculate_winning_margin(results1, results2, 'kg CO2 per km')
    return total_GWP

def determine_vehicle_year(start_year):
    if 2020 <= start_year <= 2025:
        return '2020'
    elif 2025 <= start_year <= 2030:
        return '2025'
    elif 2030 <= start_year <= 2035:
        return '2030'
    elif 2035 <= start_year <= 2040:
        return '2035'
    elif 2040 <= start_year <= 2045:
        return '2040'
    elif 2045 <= start_year <= 2050:
        return '2045'
    else:
        return '2050'

def compute_results(vehicles, size, lifetime, method, scenario, region, start_year, mileage):
    results_dict = {}
    for vehicle in vehicles:
        df_vehicle_scenario = compute_cumulative(
            PLCA(vehicle, size, lifetime, method, scenario, region, 
                 start_year=start_year, end_year=(start_year + lifetime), 
                 system_expansion=(start_year + lifetime + 1), step=5, 
                 lifecycle=200000, case=mileage, location=region), start_year
        )
        df_vehicle_scenario = df_vehicle_scenario[df_vehicle_scenario['Type'] == 'End-of-life']
        df_vehicle_scenario['kg CO2 per km'] = df_vehicle_scenario['Cumulative Value'] / df_vehicle_scenario['Total Mileage']
        results_dict[(vehicle, size, lifetime, method, scenario, start_year, mileage, region)] = df_vehicle_scenario
    return pd.concat(results_dict.values(), ignore_index=True)

def calculate_gwp(df, column_name):
    vehicle_map = {
        'transport, car, fuel cell electric,': 'FCEV',
        'transport, car, diesel hybrid,': 'HEV',
        'transport, car, battery electric,': 'BEV',
        'transport, car, plugin diesel hybrid,': 'PHEV'
    }
    
    for vehicle_type_str, vehicle_type in vehicle_map.items():
        df.loc[df['Vehicle'].str.contains(vehicle_type_str, case=True, na=False), 'Vehicle'] = vehicle_type
    
    df[column_name] = df['Cumulative Value'] / df['Total Mileage']
    print(f"Calculated {column_name} column:")  # Debug statement
    print(df[[column_name]])  # Debug statement
    mean_value = df[column_name].mean()
    return mean_value

def calculate_winning_margin(df1, df2, column_name):
    df1['Vehicle'] = 'Vehicle1'
    df2['Vehicle'] = 'Vehicle2'
    df = pd.concat([df1, df2])
    
    print("Combined DataFrame for both vehicles:")  # Debug statement
    print(df.head())  # Debug statement

    group_columns = ['Scenario', 'Region', 'Size', 'Total Mileage', 'Year', 'Type', 'Start', 'Method']
    df_grouped = df.groupby(group_columns + ['Vehicle'])
    mean_values = df_grouped[column_name].mean().unstack()
    
    print("Mean values after grouping:")  # Debug statement
    print(mean_values)  # Debug statement
    
    mean_values['Winning Margin'] = (mean_values['Vehicle1'] - mean_values['Vehicle2']) / mean_values['Vehicle2'] * 100
    df_flattened = mean_values['Winning Margin'].reset_index()
    df = df.merge(df_flattened, on=group_columns, how='left')
    return df['Winning Margin'].iloc[0]
```

In [ ]:

```
# Function call examples:
compute_gsa_analysis('arbitrary_score', 'HEV')
compute_gsa_analysis('arbitrary_score', 'BEV')
compute_gsa_analysis('arbitrary_score', 'PHEV')
compute_gsa_analysis('arbitrary_score', 'FCEV')

compute_gsa_analysis('outcome_vs_vehicle', 'BEV', 'HEV')
compute_gsa_analysis('outcome_vs_vehicle', 'PHEV', 'HEV')
compute_gsa_analysis('outcome_vs_vehicle', 'FCEV', 'HEV')

compute_gsa_analysis('outcome_within_region', 'BEV', 'HEV', 'CSA')
compute_gsa_analysis('outcome_within_region', 'PHEV', 'HEV', 'CSA')
compute_gsa_analysis('outcome_within_region', 'FCEV', 'HEV', 'CSA')

compute_gsa_analysis('outcome_within_region', 'BEV', 'HEV', 'EEU')
compute_gsa_analysis('outcome_within_region', 'PHEV', 'HEV', 'EEU')
compute_gsa_analysis('outcome_within_region', 'FCEV', 'HEV', 'EEU')
```

In [47]:

```
# Load the provided data from the files
files = [
    'GSA_BEV_Arb_20240920_075831.xlsx',
    'GSA_BEV_OutcomeVsHEV_20240925_230328.xlsx',
    'GSA_BEV_OutcomeWithinRegion_EEU_20240929_065725.xlsx',
    'GSA_BEV_OutcomeWithinRegion_CSA_20241002_073428.xlsx'
]

#    # Define light colors for shading each vehicle type
#    shading_colors = {
#        'BEV': 'tab:green',
#        'PHEV': 'tab:blue',
#        'HEV': 'tab:orange',
#        'FCEV': 'tab:pink'
#    }

# Read data from each file
dfs = [pd.read_excel(file) for file in files]

# Define the color for the bars
color = 'tab:green'

# Set up the plot
fig, axes = plt.subplots(1, 4, figsize=(8, 2), sharey=True)

# Plot the Total Order Sobol Indices for each DataFrame
for i, df in enumerate(dfs):
    df = df[df['Parameter'] != 'Number of Trajectories']
    axes[i].barh(df['Parameter'], df['Total_Order_Sobol_Index'], color=color)
    #axes[i].set_title(f'File {i+1}')
    #axes[i].tick_params(axis='x', rotation=90)

# Set common labels
#fig.suptitle('Total Order Sobol Indices')
#fig.text(0.5, 0.04, 'Parameter', ha='center')
#fig.text(0.04, 0.5, 'Total Order Sobol Index', va='center', rotation='vertical')

plt.tight_layout()
plt.savefig("A1_GSA.svg")
plt.show()
```

# 6 Time-adjusted methods plot¶

In [48]:

```
import pandas as pd
import matplotlib.pyplot as plt

# Replace 'your_file_path.xlsx' with the path to your Excel file
file_path = 'Figure4_tests.xlsx'

# Load the data into a pandas dataframe
df = pd.read_excel(file_path)

# Now we can create a pivot table to organize the data for plotting
pivot_df = df.pivot_table(index='Year', columns='Scenario', values='Cumulative Value', aggfunc='sum')

# Define line styles and markers for each scenario
line_styles = ['-', '--', '-.', ':', '-']
markers = ['o', 's', 'D', '^', 'P']

# Plotting
plt.figure(figsize=(3.7, 4))

for i, scenario in enumerate(pivot_df.columns):
    plt.plot(pivot_df.index, pivot_df[scenario], label=scenario, linestyle=line_styles[i % len(line_styles)], 
             color='#843033', marker=markers[i % len(markers)], linewidth=1, markersize=4)  # Thinner lines and smaller markers

# Adding titles and labels
plt.title('(a) Varying future scenario')
plt.xlabel('Year')
plt.ylabel('Total kg CO2')
plt.ylim(0,60000)  # Start y-axis at 0
plt.xlim(2024, 2051)  # X-axis limits for years
plt.legend(title='Scenario')

# Display the plot
plt.tight_layout()
plt.savefig("a-a4.svg")
plt.show()
```

In [49]:

```
import pandas as pd
import matplotlib.pyplot as plt

# Replace 'your_file_path.xlsx' with the path to your Excel file
file_path = 'Figure4_tests_lifetime.xlsx'

# Load the data into a pandas dataframe
df = pd.read_excel(file_path)

# Ensure the data is sorted by 'Lifetime' and 'Year' columns
df = df.sort_values(by=['Lifetime', 'Year'])

# Define the line style and marker for each Lifetime
line_style = '-.'
marker = 'D'

# Plotting
plt.figure(figsize=(3.7, 4))

# Group data by 'Lifetime' and plot each group
for lifetime, group in df.groupby('Lifetime'):
    plt.plot(group['Year'], group['Cumulative Value'], linestyle=line_style, 
             color='#843033', marker=marker, linewidth=1, markersize=4, label=f'Lifetime {lifetime}')

# Adding titles and labels
plt.title('(a) Varying Lifetime')
plt.xlabel('Year')
plt.ylabel('Total kg CO2')
plt.ylim(0, 60000)  # Set y-axis limit
plt.xlim(2024, 2052)  # Set x-axis limit

# Ensure the layout is tight for saving the figure properly
plt.tight_layout()

# Save the figure as an SVG file
plt.savefig("a-a4.svg")

# Display the plot
plt.show()
```

In [50]:

```
import pandas as pd
import matplotlib.pyplot as plt

# Replace 'your_file_path.xlsx' with the path to your Excel file
file_path = 'Figure4_tests_startyear.xlsx'

# Load the data into a pandas dataframe
df = pd.read_excel(file_path)

# Ensure the data is sorted by 'Start' and 'Year' columns
df = df.sort_values(by=['Start', 'Year'])

# Define the line style and marker for each Start year
line_style = '-.'
marker = 'D'

# Plotting
plt.figure(figsize=(3.7, 4))

# Group data by 'Start' and plot each group
for start_year, group in df.groupby('Start'):
    plt.plot(group['Year'], group['Cumulative Value'], linestyle=line_style, 
             color='#843033', marker=marker, linewidth=1, markersize=4, label=f'Start {start_year}')

# Adding titles and labels
plt.title('(a) Varying production year')
plt.xlabel('Year')
plt.ylabel('Total kg CO2')
plt.ylim(0, 60000)  # Set y-axis limit
plt.xlim(2024, 2052)  # Set x-axis limit

# Ensure the layout is tight for saving the figure properly
plt.tight_layout()

# Save the figure as an SVG file
plt.savefig("a-a4.svg")

# Display the plot
plt.show()
```

In [ ]:

```

```
